# High-fidelity tissue super-resolution imaging achieved with confocal^2^ spinning-disk image scanning microscopy

**DOI:** 10.1038/s41377-025-01930-x

**Published:** 2025-08-04

**Authors:** Qianxi Liang, Wei Ren, Boya Jin, Liang Qiao, Xichuan Ge, Yunzhe Fu, Xiaoqi Lv, Meiqi Li, Peng Xi

**Affiliations:** 1 Department of Biomedical Engineering, National Biomedical Imaging Center, College of Future Technology, Peking University, Beijing, China; 2 Airy Technologies Co. Ltd, Beijing, China; 3 School of Life Sciences, Peking University, Beijing, China

**Keywords:** Super-resolution microscopy, Biophotonics

## Abstract

Super-resolution imaging has revolutionized our ability to visualize biological structures at subcellular scales. However, deep-tissue super-resolution imaging remains constrained by background interference, which leads to limited depth penetration and compromised imaging fidelity. To overcome these challenges, we propose a novel imaging system, confocal² spinning-disk image scanning microscopy (C^2^SD-ISM). It integrates a spinning-disk (SD) confocal microscope, which physically eliminates out-of-focus signals, forming the first confocal level. A digital micromirror device (DMD) is employed for sparse multifocal illumination, combined with a dynamic pinhole array pixel reassignment (DPA-PR) algorithm for ISM super-resolution reconstruction, forming the second confocal level. The dual confocal configuration enhances system resolution, while effectively mitigating scattering background interference. Compared to computational out-of-focus signal removal, SD preserves the original intensity distribution as the penetration depth increases, achieving an imaging depth of up to 180 μm. Additionally, the DPA-PR algorithm effectively corrects Stokes shifts, optical aberrations, and other non-ideal conditions, achieving a lateral resolution of 144 nm and an axial resolution of 351 nm, and a linear correlation of up to 92% between the original confocal and the reconstructed image, thereby enabling high-fidelity super-resolution imaging. Moreover, the system’s programmable illumination via the DMD allows for seamless realization with structured illumination microscopy modality, offering excellent scalability and ease of use. Altogether, these capabilities make the C^2^SD-ISM system a versatile tool, advancing cellular imaging and tissue-scale exploration for modern bioimaging needs.

## Abstract (web summary)

C²SD-ISM integrates an SD and a DMD to form a dual confocal configuration, enabling deep-tissue super-resolution imaging with DPA-PR reconstruction.

## Graphical abstract

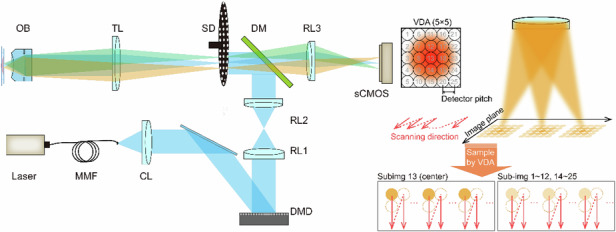

## Introduction

The advent of confocal microscopy has significantly revolutionized life sciences by enabling high-resolution three-dimensional imaging of biological specimens^1^. However, it still faces limitations when applied to deep-tissue samples. For example, stimulated emission depletion (STED) microscopy^2^, while capable of attaining better spatial resolution, suffers from resolution loss at deep tissue, which is caused by distortions of the doughnut-shaped depletion beam^3,4^. Structured illumination microscopy (SIM) is often limited by scattering-induced disruption of its stripe patterns^5–7^, resulting in significant artifacts in deep imaging. Single-molecule localization microscopy (SMLM) is also hindered by background fluorescence and increased light scattering in tissue samples^8^, which can reduce localization accuracy and distort the image structures. As an evolution of confocal laser scanning microscopy, image scanning microscopy (ISM) retains its high optical sectioning capability while achieving a $\sqrt{2}$-fold resolution improvement, which can be further improved to nearly two-fold through deconvolution^9,10^. Various implementations of ISM have demonstrated distinct advantages in imaging depth and quality when it comes to resolving complex structures in thick tissue samples^11–19^, offering a unique balance of resolution, depth, and imaging quality.

In our previous work^19^, we proposed a multi-confocal image scanning microscopy (MC-ISM) aimed at overcoming the limitations of existing tissue imaging techniques in terms of spatial-temporal resolution balance, which was accomplished by optimizing the ratio between pinhole diameter and pitch of the multifocal illumination mask, and introducing frame reduction reconstruction algorithms. However, several critical challenges persist in achieving high-fidelity imaging in deep tissues. Although the optical lock-in detection post-processing in this work effectively removes out-of-focus signals computationally, its performance relies on the configuration of pinhole diameters and spacing to avoid crosstalk. As imaging depth increases, the signal-to-noise ratio (SNR) decreases, leading to the emergence of artifacts. Furthermore, pixel reassignment (PR)-based reconstruction, derived from the core principle enabling super-resolution in ISM, usually assumes ideal point spread functions (PSFs) for both excitation and detection, without accounting for Stokes shifts or misalignments in the optical system^17^, which ultimately compromises the fidelity of reconstructed images.

Since the invention of spinning-disk (SD) confocal microscopy in 1988^20,21^, it has become a crucial imaging tool in biological research due to its excellent optical sectioning and rapid volume imaging capabilities. This technique achieves the removal of out-of-focus signals across the full field of view (FOV) by rotating a disk with an array of regularly spaced pinholes arranged in an Archimedean spiral. Furthermore, it disperses the high-intensity excitation light from single-point confocal microscopy into thousands of sub-beams, enabling low-phototoxicity imaging by achieving time-averaged exposure, which reduces the need for increased laser power while maintaining imaging efficiency.

Here, we propose a novel imaging approach, confocal² spinning-disk image scanning microscopy (C^2^SD-ISM), designed for high-fidelity deep-tissue super-resolution imaging via a dual-confocal strategy. This system integrates an SD confocal microscope, which physically removes out-of-focus signals, establishing the first confocal level. Additionally, a digital micromirror device (DMD) enables sparse multifocal illumination, while a dynamic pinhole array pixel reassignment (DPA-PR) algorithm for ISM super-resolution reconstruction, forming the second confocal level. Unlike computational methods that remove out-of-focus signals, the SD preserves the original intensity distribution as the penetration depth increases, reaching a depth of up to 180 μm. The DPA-PR algorithm effectively corrects Stokes shifts, optical aberrations, and other non-ideal conditions, achieving a lateral resolution of 144 nm and an axial resolution of 351 nm, minimizing reconstruction artifacts and achieving a linear correlation of up to 92% between the original confocal and the reconstructed result, thereby enabling high-fidelity super-resolution imaging. Further, C^2^SD-ISM can achieve high-throughput super-resolution imaging, benefiting from the high-speed SD confocal, rapid switching of the DMD, and the uniform illumination provided by the multi-mode square homogenizing fiber. Moreover, C^2^SD-ISM can be seamlessly integrated with confocal-enhanced SIM, leveraging the flexible programmability of the DMD, offering excellent scalability and ease of use. All these capabilities make the C^2^SD-ISM system a versatile tool for advancing cellular imaging and tissue-scale exploration, meeting the demands of modern bioimaging.

## Results

### Hardware implementation

**Fig. 1:**
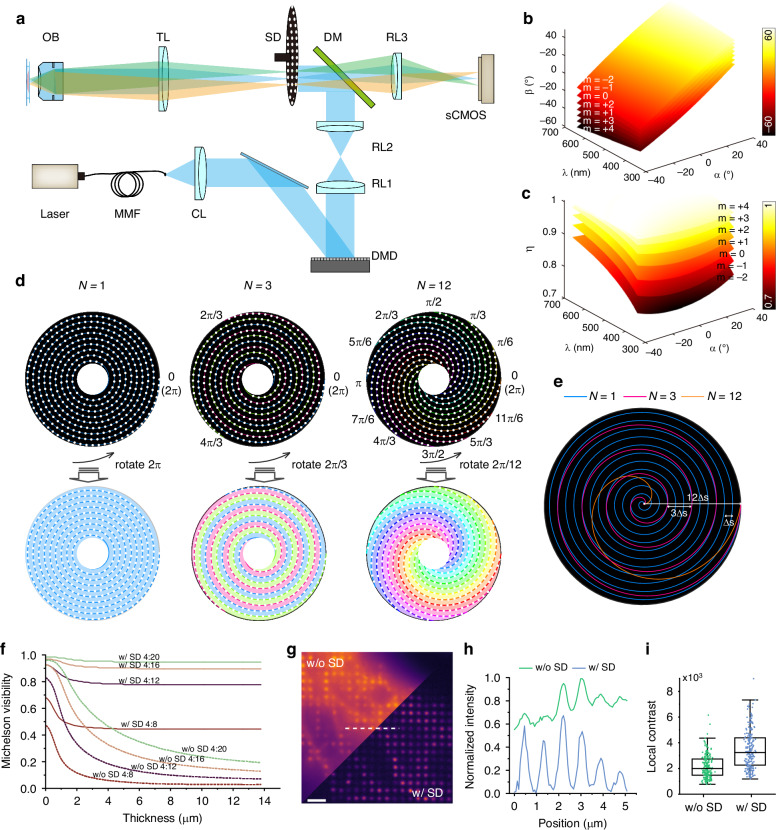
Optical design and imaging performance evaluation of the C^2^SD-ISM system. **a** Optical configuration of C^2^SD-ISM. MMF multi-mode fiber, CL collimating lens, DMD digital micromirror device, RL1, RL2, RL3 relay lenses, DM dichroic mirror, SD spinning-disk, TL tube lens, OB objective lens. **b** Quantitative relationship between exit angle β, wavelength λ, and incident angle α. **c** Quantitative relationship between diffraction efficiency η, wavelength λ, and incident angle α. **d** Diagram showing the effect of different *N* values. As *N* increases, the pinholes are distributed across multiple concentric spirals, with 2π/*N* covering the entire FOV. **e** Comparison of Archimedean spiral overlays for different *N* values. **f** Impact of SD on imaging visibility at different sample thicknesses and with different DMD masks. **g** Comparison of DMD-generated multifocal illumination results on a mouse kidney section sample with and without the SD. Scale bar: 2 μm. **h** Normalized fluorescence intensity profile along the dashed line in (**g**). **i** Box plot of local contrast of fluorescence intensity with and without SD

The optical configuration of the C^2^SD-ISM system is depicted in Fig. 1a. A high-speed SD is placed on the sample conjugate plane, realizing the real-time removal of out-of-focus signals. This reduces the background interference that would otherwise affect the subsequent reconstruction process. Multifocal illumination for super-resolution imaging is generated by a DMD, which offers more stable stepping than utilizing the pinhole array and galvanometer for the generation and scanning of multifocal spots in our previous work. Here, the sample focal plane, the SD pinhole array, the DMD, and the sCMOS sensor plane are conjugated. The illumination is jointly modulated by both DMD and the SD; and the excited fluorescence is filtered by the SD pinhole array before reaching the sCMOS camera.

By selecting a high-power multi-mode laser as the illumination source and using a square homogenizing fiber, speckle-free and uniform illumination well-matched to the DMD plane is achieved. Though the DMD functions as a projection device, its array structure exhibits the properties of a two-dimensional blazed diffraction grating (Fig. S1)^22^, resulting in the wavelength-dependent maximum diffraction efficiency and variations in direction of the outgoing light, which in turn causes FOV discrepancy and uneven illumination during multicolor imaging. To address this issue, we systematically analyzed the dependence of diffraction efficiency and exit angle on both wavelength and incidence angle (Note S2 and Fig. 1b, c), in order to determine the optimal incidence angle for multicolor imaging. Setting the incidence angle to 26.3° achieves over 95% diffraction efficiency for tri-color imaging (561 nm, 488 nm, and 405 nm), with corresponding exit angles of −6.9°, −2.5°, and 2.5°. The resulting FOV sacrifices are negligible relative to the 1024 × 1024 target size.

In super-resolution imaging, sample structures need to be modulated by multifocal illumination. The image formation can be expressed as:

1 $${I}_{i}=[{\rm{Obj}}({\bf{r}},z)\cdot { {\mathcal I} }_{i}({\bf{r}},z)]{\otimes }_{3D}{{\rm{PSF}}}_{{\rm{sys}}}({\bf{r}},z)$$

Where *I*, Obj, ${\mathcal I}$ and PSF_sys_ represent the acquired raw frame, sample structure, structured illumination generated by the DMD, and the system’s PSF, respectively. The subscript i corresponds to the *i*-th frame, **r** and *z* denote the 3D coordinates, and ${\otimes }_{3D}$ represents the 3D convolution operation. The DMD mask used to generate the multifocal illumination is designed as a periodic lattice, with each element consisting of a square aperture formed by 4 × 4 “ON” state DMD pixels. Given that each DMD pixel measures 5.4 μm, the excitation spot size on the sample plane is 216 nm under a 100× objective. The multifocal pattern is shifted by two DMD pixels at a time, corresponding to a 108 nm step size on the sample plane, achieving a sampling rate twice the diffraction-limited. Without the SD (w/o SD), the system operates in wide-field detection mode, where PSF_sys_ corresponds to the detection PSF. With the SD (w/ SD), PSF_sys_ transforms into a confocal PSF, characterized by a narrower axial shape, showing better axial resolution. Figure 1f and Movie S1 demonstrate that the system with SD is compatible with thicker samples and closer excitation spot spacing. Therefore, we can employ a 4:12 (the ratio of squared aperture side length to periodicity) mask, requiring only 6 × 6 raw images (Figs. S2 and S9). The number of raw frames required for a complete scan is reduced to 1/6 times that of MSIM^11^. The timing sequence for image acquisition using the DMD is illustrated in Fig. S3.

### Design and validation of the SD pinhole pattern

The SD, fabricated using an electron-beam lithography and etching process, features pinholes arranged along an Archimedean spiral. Each 2π angular increment maintains a constant radial step Δ*s* equal to the pinholes spacing, ensuring uniform illumination across the full FOV^20,21,23,24^ (Note S3 and Fig. 1d, e).

During a single-frame illumination period of the DMD, the SD must fully cover the entire FOV uniformly. With a single Archimedean spiral design and a rotation speed of 5000 rpm, it takes a period of 12 ms to complete one full FOV coverage, which significantly limits the overall imaging speed. To address this issue, we developed a multi-concentric spiral design (*N* > 1), comprising *N* sub-spirals. Compared to the single-spiral (*N* = 1) design, the radial stepping distance for each sub-spiral is expanded to *N* times the original value per 2π rotation (Fig. 1d and Movie S2). Each sub-spiral is rotated by 2π/*N* relative to the others, resulting in complete overlap. Consequently, the full FOV is divided into *N* sections, enabling parallel scanning. This design theoretically increases the uniform illumination speed across the full FOV by a factor of *N* compared to the single-spiral configuration. This reduces the adjustable exposure time interval to 1/*N* of the original, which simplifies the synchronization between exposure time and SD rotation speed, facilitating stable and efficient operation.

Moreover, increasing *N* not only enhances scanning efficiency but also improves the system’s robustness to the center aligning requirement. As shown in Fig. S4 and Movie S2, even with a center offset of 500 μm caused by mechanical manufacturing, the *N* = 12 design maintains uniform illumination across the FOV, whereas the *N* = 1 design exhibits significant nonuniform illumination. However, excessively increasing *N* can result in the radial spacing between adjacent pinholes exceeding the Nyquist sampling criterion. According to Eq. S31, both a larger *N* value and a smaller radial position result in a larger radial step size. Therefore, in SD pattern design, maximizing the use of the disk’s outer areas while keeping *N* within an appropriate range can prevent excessive radial stepping. An *N* value of 12 was selected to optimally balance between robustness and radial sampling.

For the SD, sectioning performance is determined by the pinhole diameter and spacing. Decreasing the pinhole diameter effectively blocks out-of-focus signals, improving both lateral and axial resolution, while increasing the spacing minimizes signal crosstalk. However, these adjustments reduce the disk’s fill factor and light throughput, resulting in a reduced SNR. A pinhole diameter-to-spacing ratio of 50 μm:250 μm was employed in our experiments to balance these trade-offs.

The defocus signal removal capability of the SD was validated using a U2OS cells sample and a mouse kidney section. As shown in Fig. S5, the setup incorporating the SD effectively revealed out-of-focus signals, providing clearer sample details compared to the setup without it, resulting in approximately three-fold improved contrast. Further assessment under DMD-generated multifocal excitation demonstrates that the introduction of the SD enhances the SNR of the image and the local contrast (LC) at individual spots (Fig. 1g–i). Consequently, the multifocal pattern becomes clearer, ensuring higher fidelity in subsequent algorithmic reconstruction.

### DPA-PR reconstruction using a virtual detector array (VDA)

Conventional ISM reconstruction typically involves optically or computationally reducing the spot size by half or doubling the spot spacing, an operation known as PR^9,25^, where detected signals are shifted back to their original positions. The standard scaling factor of 1/2 assumes an idealized system in which the excitation and detection PSF are Gaussian-like and identical, with no Stokes shift or distortion. However, these assumptions often do not hold in practical implementations. To address these limitations, adaptive pixel reassignment (APR) has been proposed^17^, where each pixel in the detector array captures images of the same sample region from different perspectives. By aligning these images spatially through cross-correlation registration, adaptive scaling is achieved instead of the fixed 1/2. However, APR relies on a scan-and-descan process to keep the focal point stationary on the detector array, making it suitable only for single-point scanning configurations (Note S1). In the C^2^SD-ISM system, this constraint is altered as the spot moves across the camera’s target surface during sample scanning.

**Fig. 2:**
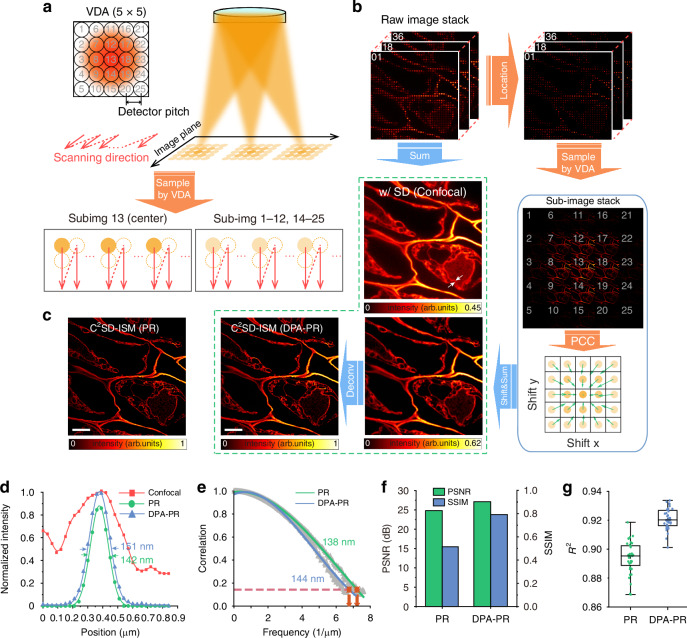
DPA-PR reconstruction process and results. **a** Schematic diagram of VDA construction and sampling. **b** Schematic diagram of the DPA-PR reconstruction process. The PCC results show the estimated shift vectors, with green arrows indicating the direction and size of spatial offset, and the shade of the yellow circles representing the SNR of sub-images. The maximum gray value of DPA-PR-reconstructed C^2^SD-ISM image was normalized to 1 to generate the intensity scale bars. Scale bar: 5 μm. **c** PR reconstruction results of the same region. Scale bar: 5 μm. **d** Intensity profiles of the three modalities indicated by the white arrow in (**b**). **e** Resolution comparison obtained by FRC analysis under PR and DPA-PR. **f** Column chart of PSNR and SSIM comparison between PR and DPA-PR. **g** Box plot of the coefficient of determination (*R*^2^) comparison between PR and DPA-PR

To apply APR to this dynamic setup and achieve higher fidelity in reconstruction, we proposed a DPA-PR approach^17,26^. This method constructs a 5 × 5 VDA by positioning its center pixel along the excitation axis and synchronizing its movement with the sample scan, with a spacing equal to the scanning step (Fig. 2a). Under multifocal excitation, each focus spot has an associated VDA, with each VDA element’s intensity obtained by interpolating from the camera’s recorded intensity values. By rearranging the intensity values of each VDA element based on the spot position and scan order, 25 confocal sub-images are extracted from the raw image stack. Each sub-image contains identical structural information but differs in spatial shifts and SNR. The DPA-PR reconstruction process, as applied to a mouse kidney tissue section, is illustrated in Fig. 2b and Movie S3 and involves three main steps: (1) excitation optical axis array localization, (2) VDA sampling, and (3) registration and superposition. The final C^2^SD-ISM image is further deconvolved to realize a two-fold resolution improvement.

To locate the excitation axis, background noise is removed from each image by suppressing the zero-frequency component along the scan direction. The basis vectors of the illumination lattice are then determined through Fourier spectrum analysis, and the lattice’s absolute position is calculated by averaging the offset between lattice points generated by basis vectors and actual illumination peaks (Fig. S6). This process yields a precise scan step of ~0.2 AU (1.6 × 65 nm @ 640 nm and 1.49 NA). This step serves as the spacing for constructing the VDA, allowing the image to be sampled and reorganized into 25 confocal sub-images. Phase cross-correlation (PCC)^27^ between edge sub-images and the central sub-image is used for precise registration, and the result of the theoretical $\sqrt{2}$-fold resolution improvement is obtained through direct superposition. After deconvolution, a two-fold resolution improvement is achieved (Fig. S7a). Detailed reconstruction steps are provided in Note S4.

The maximum gray value of DPA-PR-reconstructed C^2^SD-ISM was normalized to 1 to generate the intensity scale bars. As evident from Fig. 2b, registration and superposition, followed by deconvolution, enhance the maximum signal intensity of the image, thereby improving the SNR. Compared to confocal imaging, DPA-PR significantly improves the resolution, with deconvolution enabling a total two-fold resolution enhancement, allowing visualization of structures as small as 151 nm (Figs. 2d and S7b). DPA achieves a resolution comparable to that of conventional PR reconstruction^28^ (Fig. 2c–e). Additionally, DPA-PR accounts for non-ideal factors such as system aberrations and Stokes shifts, with offsets encoded in the spatial positioning of sub-images instead of using a fixed two-fold scaling factor. This approach is more consistent with the physical model of ISM, resulting in higher fidelity in the reconstruction outcomes. Using diffraction-limited confocal images as references, the reconstruction results of DPA-PR exhibit better peak signal-to-noise ratio (PSNR) and structural similarity (SSIM) index (Fig. 2f). Image quality was also assessed with NanoJ-SQUIRREL^29^ (Fig. S7c), revealing that both DPA-PR and PR achieved resolution-scaled Pearson coefficients (RSP) above 0.9, indicating high fidelity. However, DPA-PR displayed fewer artifacts in areas with large intensity changes and achieved a lower resolution scaled error (RSE). To further evaluate linear correlation, signal regions from the reconstructed images were sampled randomly, and the coefficient of determination (*R*^2^) was calculated between the super-resolution images degraded by the resolution scaling function (RSF) and the confocal images. DPA-PR demonstrated superior linear correlation with a mean *R*^2^ value of 0.92, exceeding that of PR (Fig. 2g). Compared to other open-source ISM reconstruction algorithms, such as PR based on frequency domain points positioning and iterative algorithms based on joint Richardson-Lucy deconvolution, DPA-PR also demonstrates the best linear correlation, comparable to APR (Fig. S8).

## The impact of the SD on enhancing 3D resolution and reconstruction fidelity

**Fig. 3:**
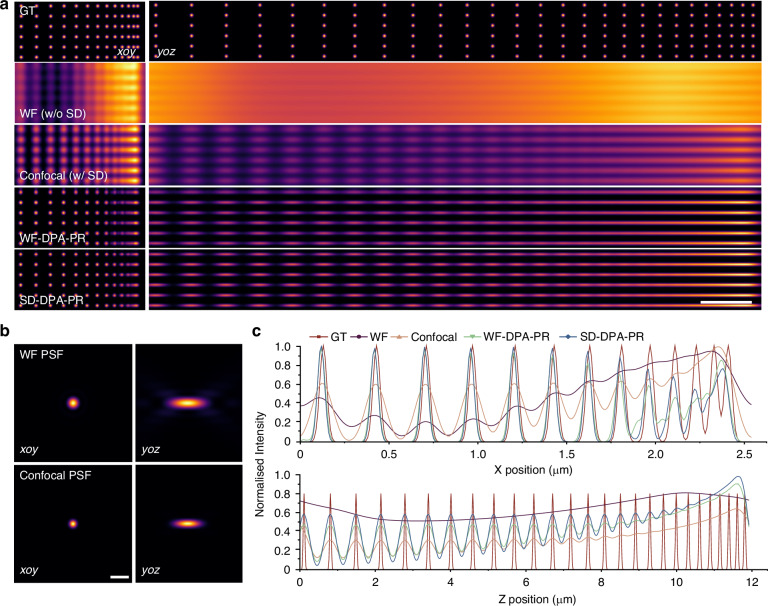
The impact of the SD on enhancing 3D resolution. **a** Simulation 3D results (*xoy* and *yoz*) using the cube of spherical beads as the 3D synthetic dataset. Scale bar: 1 μm. **b** Simulated wide-field and confocal PSFs with cross-sectional views in the *xoy* and *yoz* planes. Scale bar: 400 nm. **c** Intensity profiles along the *x*- and *z*-axes through the centers of the beads

We evaluated the role of the SD in enhancing 3D resolution and enabling high-fidelity super-resolution reconstruction through simulation experiments and biological tissue imaging. In the simulations, a synthetic cube of spherical beads (Fig. S10a) was used to quantitatively evaluate the resolution improvement achieved by DPA-PR and SD (Note S5). Wide-field (w/o SD) and confocal (w/ SD) imaging were simulated by convolving with a 3D PSF (PSF parameters: emission wavelength 500 nm, NA 1.49, Fig. 3b). Raw frames were then generated using multifocal illumination, and the DPA-PR algorithm was applied to obtain super-resolution results, producing WF-DPA-PR and SD-DPA-PR reconstructions (Figs. 3a and S10b–e). By comparing reconstruction results, it was observed that the DPA-PR algorithm can effectively enhance the resolution by a factor of $\sqrt{2}$ regardless of the presence of SD. Moreover, the introduction of SD further improved the DPA-PR reconstruction, resulting in a 20 nm improvement in lateral resolution and a 60 nm improvement in axial resolution (Fig. 3c).

**Fig. 4:**
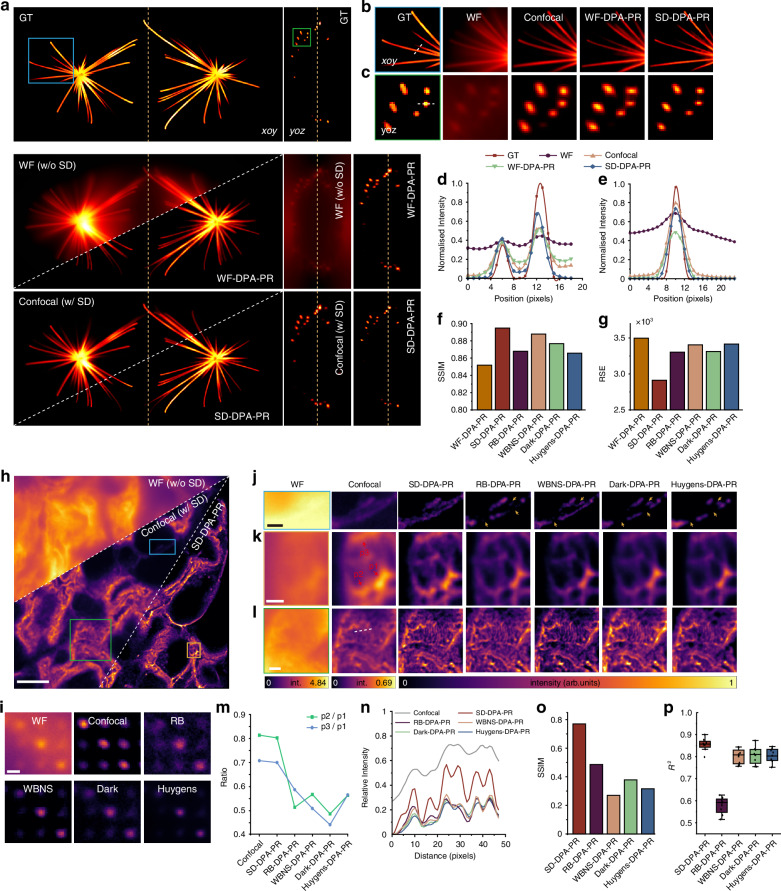
The impact of the SD on enhancing reconstruction fidelity. **a** Simulation 3D results (*xoy* and *yoz*) using microtubules as the 3D synthetic dataset. Ground truth (GT) images, wide-field (w/o SD), and confocal (w/ SD) simulations, as well as corresponding super-resolution reconstructions using the DPA-PR algorithm (WF-DPA-PR and SD-DPA-PR). **b**, **c** Magnified views of sub-regions in the *xoy* (**b**) and *yoz* (**c**) planes, showing the 3D reconstruction structural differences between the ground truth image and the four imaging modes. The two regions correspond to the locations marked by the blue and green rectangular boxes in (**a**). **d**, **e** Intensity profiles along the white dashed lines in (**b**, **c**), comparing the GT image with the four imaging modes. **f**, **g** SSIM and RSE comparisons of DPA-PR reconstructions based on wide-field, confocal, and various computational background removal methods, including RB, WBNS, Dark, and Huygens. **h** Imaging results of a highly scattering mouse kidney section under wide-field, confocal, and SD-DPA-PR. Scale bar: 10 μm. **i** Comparison of the original point distribution patterns under wide-field, confocal, and different computational background removal algorithms. Scale bar: 0.5 μm. **j**–**l** Comparison of magnified views from wide-field, confocal, and DPA-PR reconstruction results based on SD defocus removal and other computational background removal methods. The three representative regions correspond to the locations indicated by the blue, brown, and green rectangular boxes in (**h**). Scale bars are 2 μm, 1 μm, and 2 μm, respectively. **m** Comparison of grayscale intensity ratios at the red arrow-indicated positions in (**k**). **n** The intensity distribution corresponding to the white dashed line in (**l**). **o**, **p** SSIM and *R*² comparisons of DPA-PR reconstructions based on SD defocus removal and various computational background removal algorithms

Furthermore, an open-source dataset of synthetic microtubules^30^ was used as ground truth to simulate the impact of SD on imaging and super-resolution reconstruction (Fig. 4a). DPA-PR significantly improved the three-dimensional clarity of the samples. However, without the SD, overlapping information from different depths severely hindered structural analysis. The magnified sub-region in the *xoy* (Fig. 4b) and *yoz* (Fig. 4c) planes was shown and analyzed at positions marked by white dashed lines. The results showed that SD-DPA-PR presented the finest details of both lateral (Fig. 4d) and axial structures (Fig. 4e). In addition, we compared the physical defocus removal capability of the SD with two publicly available computational background removal algorithms—rolling ball (RB)^31^ and wavelet-based background and noise subtraction (WBNS)^32^—as well as iterative deconvolution using Huygens software and the Dark-based optical sectioning algorithm^33^ developed by our group (Fig. S11a, b). Compared with the computational background removal methods, the SD physical defocus removal not only preserved the sample’s structural information more effectively (Fig. S11c) but also achieved the highest SSIM and lowest RSE in quantitative comparisons (Fig. 4f, g). These findings underscore the critical role of the SD in suppressing defocus signals and achieving high-fidelity super-resolution reconstruction.

To further validate the effectiveness of SD and DPA-PR in real biological samples, we performed experiments on 12-μm-thick mouse kidney tissue sections. Results demonstrated that SD-DPA-PR exhibited the best fidelity (Fig. S12a–d) and resolution (Fig. S12e). As the sample depth increases, scattering signals and background defocus signals become more severe. When super-resolution reconstruction is performed directly on wide-field images without effective defocus removal, issues such as a reduced lattice illumination visibility (Fig. S13a, h), shift vector estimation error (Fig. S13b, i), and inconsistent global background thresholds (Fig. S13c) arise, leading to significant artifacts and signal loss in the final reconstruction (Fig. S13d–g). With the introduction of the SD, defocus signals were progressively reduced, resolution was enhanced, and the clarity of sample details significantly improved, from wide-field imaging to confocal imaging and ultimately to SD-DPA-PR (Fig. 4h).

Meanwhile, applying different background removal algorithms to wide-field images showed that although some background could be removed, these algorithms alter the original illumination spots’ intensity distribution, potentially leading to issues such as signal loss, incomplete background removal, excessive smoothing, and decreased fidelity of the reconstruction results (Fig. 4i). Three representative regions were selected for magnification. In wide-field imaging, the repeated accumulation of out-of-focus signals led to a significantly higher maximum signal intensity compared to that acquired under confocal imaging (Fig. 4j–l). In the weaker signal region, SD-DPA-PR preserved the most complete sample information, while the background removal algorithms incorrectly removed some weak in-focus signals, resulting in discontinuous structures in the reconstruction as indicated by the yellow arrow in Fig. 4j. In the second region (Fig. 4k), ratio analysis of three points marked by red arrows revealed that SD-DPA-PR maintained a linear intensity distribution consistent with confocal imaging, whereas the other methods exhibited inconsistent bright-dark variations (Fig. 4m). In the third, a more complex region (Fig. 4l), the intensity distribution curve along the dashed line showed that the SD defocus removal-based reconstruction outperformed the other methods in terms of intensity linearity and structural contrast (Fig. 4n). Quantitative analysis further confirmed that SD-DPA-PR achieved the highest SSIM (Fig. 4o) and best linear correlation (Fig. 4p).

The combination of SD confocal and the DPA-PR algorithm demonstrated exceptional performance in super-resolution reconstruction of highly complex samples. By physically eliminating defocus signal interference, the method significantly enhanced both lateral and axial resolution while providing high-fidelity input data for reconstruction.

## Super-resolution imaging of biological tissues

**Fig. 5:**
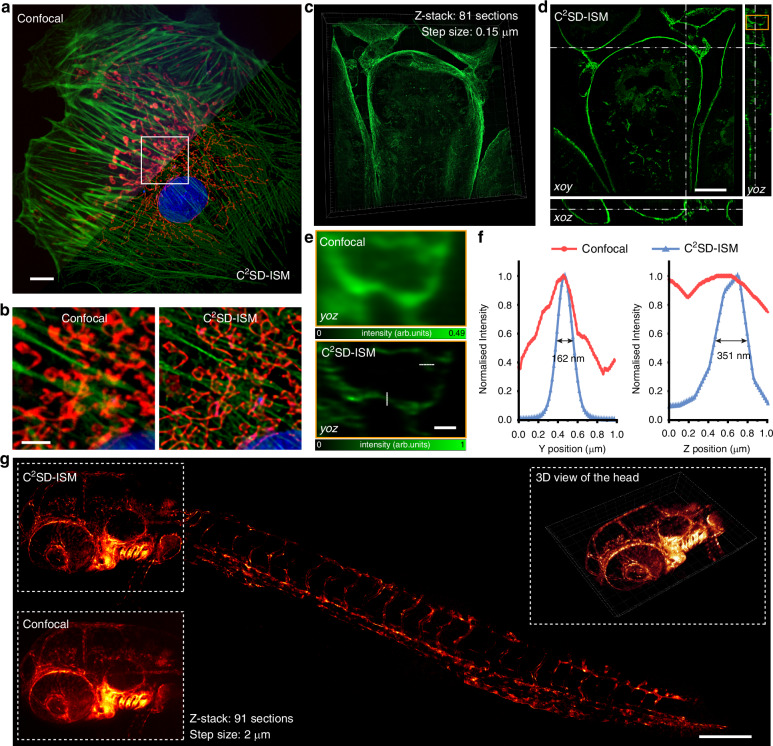
Super-resolution imaging of biological tissues using the C^2^SD-ISM system. **a** Validation of the multi-color imaging capability of C^2^SD-ISM in BPAE cells. Scale bar: 5 μm. **b** The magnified result of the white rectangular box in (**a**) shows the imaging differences between confocal and C^2^SD-ISM. Scale bar: 2 μm (**c**) 3D imaging of a mouse kidney section (Z-stack: 81 sections, step size: 0.15 μm) acquired with the C^2^SD-ISM system. **d** Orthogonal display of 3D imaging results of mouse kidney section in C^2^SD-ISM system. Scale bar: 10 μm. **e** Comparison of confocal and C^2^SD-ISM images of the orange boxed region in (**d**). Scale bar: 1 μm (**f**) Line profiles of intensity along the white dashed line in (**e**). **g** 3D maximum intensity projection of the whole-body vasculature in zebrafish with a total imaging depth of 180 μm. Insets show the confocal results (bottom left) and a 3D view (top right) of the zebrafish head vasculature, corresponding to the same region as the top left. Scale bar: 200 μm

We validated the multi-color imaging capability of C^2^SD-ISM on tri-color labeled samples, with mitochondria labeled by MitoTracker Red CMXRos, F-actin stained with Alexa Fluor 488 phalloidin, and nuclei labeled with DAPI. There were no differences in the FOV uniformity across different wavelength channels (Fig. 5a). The magnified images further highlighted that the C^2^SD-ISM results provided clearer imaging quality compared to confocal imaging (Fig. 5b). The imaging results of microtubules in fixed cells exhibited a similar trend (Fig. S14). It should be noted that although the absorption and scattering properties of biological samples are wavelength-dependent^34^ and can affect imaging depth, in the case of thin cellular specimens where imaging is dominated by single or few scattering events, the influence of wavelength on imaging depth becomes negligible.

Combining the SD with DPA-PR reconstruction, the C^2^SD-ISM system demonstrates superior 3D super-resolution imaging capabilities. We performed 3D imaging of a mouse kidney section sample over a volume of 66.5 μm × 66.5 μm × 12 μm with an axial step size of 150 nm (Fig. 5c, d). With precise optical sectioning, both surface contours and internal structural details of the kidney are distinctly observed, and the further magnified *yoz* images reveal clear axial details (Fig. 5e). The system achieved nearly two-fold improvements in lateral and axial resolution, reaching 162 nm and 351 nm, respectively (Fig. 5f).

Another key advantage of the C^2^SD-ISM system is its high throughput, making it particularly well-suited for high content imaging (HCI). Traditional HCI is restricted by the diffraction limit, which limits the ability to observe fine subcellular structures. The C^2^SD-ISM system overcomes these limitations, achieving ultra-large FOV super-resolution imaging with a two-fold resolution improvement by stitching adjacent fields of view. We performed 3D stitched imaging of the endothelial/lymphatic of EGFP-labeled zebrafish using a 10× objective lens, covering a volume of 2.91 mm × 1.26 mm × 0.18 mm. Figure 5g and Movie S4 present a detailed maximum intensity projection of the zebrafish head vasculature, where the C^2^SD-ISM achieves significant resolution enhancement compared to conventional confocal imaging, revealing fine details that are difficult to discern in confocal images and producing sharper vascular structures. The top-right corner of Fig. 5g provides a 3D rendering of the head part, highlighting the depth and clarity achieved by the system. The main part of Fig. 5g demonstrates whole-body imaging of the zebrafish achieved by seamless FOV stitching. Compared to the confocal imaging results obtained with a 20× 0.8 NA objective lens (Fig. S15), it can be demonstrated that super-resolution reconstruction using a low magnification and low NA lens achieves resolution and clarity comparable to confocal imaging with a high magnification and NA lens. Additionally, the low magnification objective enables a larger FOV, reducing the number of frames required for field stitching. This extended FOV capability showcases the high-throughput potential of the C^2^SD-ISM system, enabling rapid acquisition of super-resolution images over large areas, making it an ideal tool for large-scale biological studies. To demonstrate the imaging advantages of C^2^SD-ISM more clearly, a performance comparison with other ISM techniques based on digital reconstruction is provided in Note S6 and Table S1.

### Projection-based SIM modality of the C^2^SD-ISM system

Leveraging the flexible pattern control provided by the DMD, the C^2^SD-ISM system supports multiple super-resolution imaging modalities. Loading stripe-patterned masks onto the DMD, it enables projection-based SIM, enhancing its versatility for biological tissue imaging. The stripe mask period is set to 8 DMD pixels, corresponding to a 432 nm period on the sample plane under a 100× objective, achieving a 1.6-fold frequency expansion with 640 nm excitation. While traditional 2D-SIM, based on interference-generated patterns, can reach nearly two-fold frequency expansion, projection-based SIM is limited by the modulation transfer function, which attenuates high-frequency signals. When the stripe period is too small, strip contrast declines rapidly, limiting resolution improvement. However, this limitation also enhances optical sectioning, as the projected stripes are mainly visible near the focal plane and fade as defocusing occurs, improving optical sectioning capacity in tissue imaging.

**Fig. 6:**
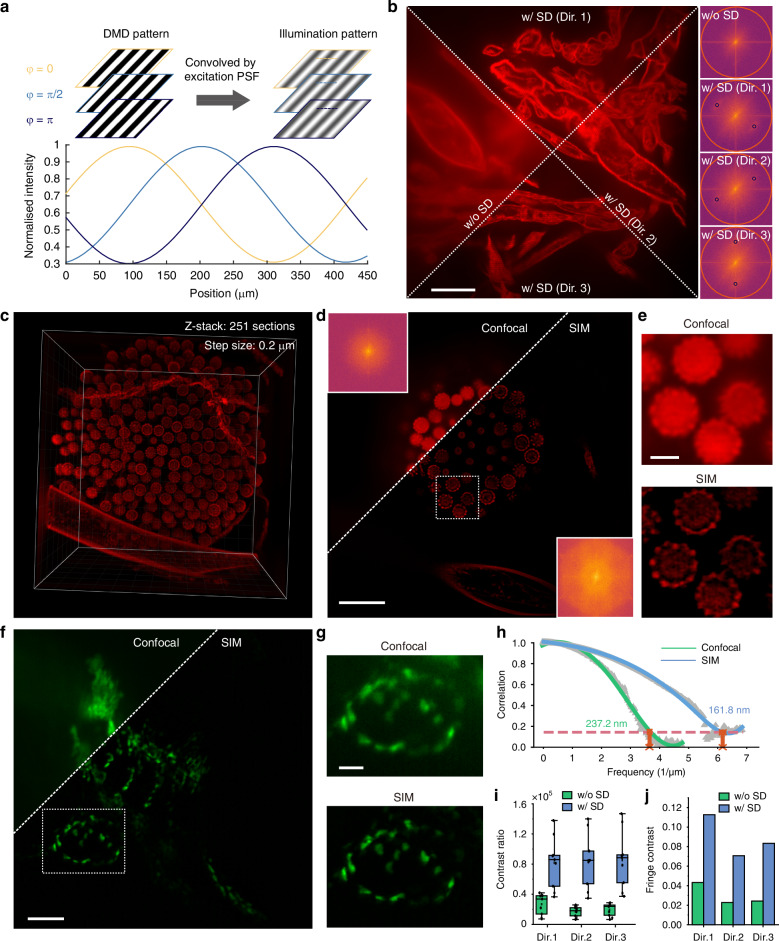
Principle and results of projection-based SIM modality of C^2^SD-ISM. **a** Stripe pattern generated by the DMD with an 8-pixel period, displayed with phase shifts of 0, π/2, and π, and their corresponding illumination profiles on the sample plane. The normalized intensity profile below shows the phase shifts. **b** Comparison of biological tissue imaging without and with the SD in three orientations (Dir. 1, Dir. 2, Dir. 3). Stripe pattern visibility is enhanced at depth with SD applied, as shown in the right frequency spectra. Scalebar: 10 μm. **c** 3D projection of a mold sample over a 66.5 × 66.5 × 50 μm volume imaged with SD-SIM, achieving a total imaging depth of 50 μm. **d** Comparison of confocal and SIM imaging of the mold sample, with insets in the top left and bottom right displaying the respective frequency spectra. Scalebar: 10 μm. **e** Detailed view of the mold sample within the area indicated by the white-dashed box in (**d**), comparing confocal and SIM imaging. Scalebar: 2 μm. **f** Comparison of confocal and SIM imaging of the EGFP-labeled zebrafish epicardial tissue. Scalebar: 25 μm. **g** Detailed view of the zebrafish epicardial tissue within the area indicated by the white-dashed box in (**f**), comparing confocal and SIM imaging. Scalebar: 10 μm. **h** Resolution comparison obtained by FRC analysis under Confocal and SIM. **i** Box plot of contrast ratio measurements of SIM in three directions with and without SD setup. **j** Column chart of SIM fringe contrast comparison in three directions with and without SD setup

To achieve uniform field illumination, SIM requires phase shifts of π/3 and 2π/3. However, implementing these shifts would require a DMD mask period of at least 6 or 12 pixels. The 6-pixel period, with a spatial frequency at 0.8 times the system cutoff, suffers from unacceptable reduction in stripe contrast, while the 12-pixel period yields only a 1.3-fold frequency expansion, limiting the resolution. To overcome these limitations, we applied a two-pixel shift to the stripe pattern, achieving π/2 and π phase shifts (Fig. 6a) and utilizing high-fidelity SIM (HiFi-SIM)^35^ for super-resolution reconstruction. The HiFi-SIM effectively minimizes artifacts and attenuates high-order frequency components arising from non-uniform illumination, yielding a HiFi-SIM reconstruction.

The introduction of the SD significantly extends the penetration depth of the stripe pattern. As shown in Fig. 6b, the projected stripe pattern is almost invisible without the SD, but is clearly observed on the sample plane with the SD at the same depth, with corresponding modulation orders visible in the frequency spectrum. By calculating the contrast ratio (CR) and fringe contrast (FC) in each direction of SIM with and without SD (Fig. 6i, j), the advantage of the SD in removing out-of-focus signals and improving imaging quality is further validated.

This improvement allowed us to perform 3D imaging of a mold sample over a volume of 66.5 × 66.5 × 50 μm (Fig. 6c and Movie S5), well beyond the penetration depth typically achievable with projection-based SIM, as well as interferometric 2D SIM^36^ and 3D SIM^37^. At a depth of 17 μm, a comparison between confocal and SIM imaging shows that the SD-based SIM delivers excellent sectioning capability and achieves a 1.68-fold improvement in resolution (Fig. 6d, h). The magnified view in Fig. 6e clearly reveals the distinctive morphological features of the fungal sporangium. To further highlight the versatility of the method, EGFP-labeled zebrafish epicardial tissue was imaged using a 20× objective lens. Compared to confocal imaging, SIM imaging achieved improved contrast and structural clarity (Fig. 6f), revealing finer subcellular structures with enhanced resolution (Fig. 6g).

## Discussion

Our study demonstrates advancements in high-fidelity imaging capabilities offered by the C^2^SD-ISM system, providing super-resolution imaging for detailed subcellular structures and deep tissue imaging. By integrating SD with DPA-PR, the C^2^SD-ISM system effectively overcomes common limitations of conventional confocal and other super-resolution microscopy, such as resolution limitation, reconstruction artifacts, background interference, and restricted penetration depth. First, the SD physically eliminates out-of-focus signal interference, enabling more precise focal localization and significantly enhancing point signal clarity. Compared to methods that rely solely on algorithmic defocus removal, the SD preserves the true spatial distribution of point signals, providing higher-quality input data for the DPA-PR reconstruction. Second, DPA-PR further refines imaging performance by accounting for PSF variations due to positional offsets, Stokes shift, and optical aberrations, achieving spatially accurate reconstructions that closely align with the ISM theoretical model. Lastly, each VDA element measures only 0.2 AU, forming an almost closed confocal pinhole configuration compared to the typical 1 AU pinhole in conventional confocal systems, which enhances resolution effectively.

It is worth noting that although the introduction of the SD effectively enhances imaging contrast, its limited fill factor also reduces the excitation power, thereby decreasing the total detected signal intensity. While the improved contrast benefits the DPA-PR reconstruction, the attenuated signal strength may diminish the SNR and spatial detail in edge confocal sub-images. To address this, future improvements may involve the use of a stripe-patterned disk or a dual-disk configuration integrated with a microlens array to enhance excitation efficiency. Incorporating deep learning-based denoising algorithms^38^ could further refine the quality of confocal sub-images before registration and integration, ultimately enabling higher-fidelity super-resolution reconstruction.

Additionally, optimizing the DMD incident angle ensures uniformity and efficiency in multi-color imaging. The flexible control over excitation patterns provided by the DMD enhances the system’s adaptability, allowing the C^2^SD-ISM to incorporate SIM.

The C^2^SD-ISM system also demonstrates significant advantages in high-throughput imaging, as shown in the 3D reconstruction of zebrafish vasculature, highlighting its potential for whole-tissue and large-area sample imaging. The system achieved a 3D imaging depth of 50 μm in mold samples, significantly exceeding the typical penetration depth of traditional projection-based SIM, underscoring its potential for visualizing complex internal structures within biological samples.

Looking ahead, the C^2^SD-ISM system’s design offers significant potential for future developments, particularly for advanced imaging applications in deeper tissue environments. Integrating adaptive optics could enhance the system’s capability to correct sample-induced aberrations^39^, allowing for more precise imaging in complex biological structures and providing higher fidelity in deeper tissues. In addition, employing long-wavelength fluorophores^34^ (e.g., in the red or near-infrared range) can reduce scattering and autofluorescence, thereby increasing penetration depth and signal-to-background ratio. Moreover, nonlinear excitation strategies such as two-photon absorption^40^, especially when combined with temporal focusing^41^, can confine excitation to the focal plane and provide intrinsic optical sectioning in thick samples. These approaches are compatible with the C^2^SD-ISM framework and represent a promising direction for subsequent integration into deep-tissue imaging applications. Furthermore, future developments in computational reconstruction, including machine learning-based algorithms^42^, could refine spatial resolution, reduce the required number of raw data frames, and shorten reconstruction time, enabling even faster and more accurate reconstructions that can respond to specific biological processes in real time.

Another promising direction is leveraging the adaptive illumination capabilities of the C^2^SD-ISM system, especially for observing transient biological processes in live cells. By leveraging the programmable illumination of the DMD to only activate regions of interest and optimizing illumination strategies to further reduce phototoxicity and photobleaching, the system could support long-term, super-resolution imaging of dynamic events, such as mitochondrial fission and fusion^43^, intracellular trafficking, and neural activity. Such enhancements would be valuable for studies requiring both high temporal resolution and minimal cellular perturbation. Furthermore, combining the method with fluctuation-based super-resolution techniques may offer additional improvements in spatial resolution^44^.

In summary, the C^2^SD-ISM system combines the advantages of both worlds: the optical sectioning capability of SD confocal microscopy, and the super-resolution achieved through parallel image scanning microscopy. The fast, programmable switching capability of the DMD system effectively expands the C^2^SD-ISM system to projection SIM and adaptive illumination as well. It therefore provides a robust and flexible platform for super-resolution imaging, effectively addressing limitations in conventional microscopy and offering new pathways for biological exploration, delivering insights into cellular structures and dynamics in deep tissue imaging, which can effectively deepen our understanding of complex biological systems.

## Materials and methods

### C^2^SD-ISM system

The C^2^SD-ISM system was mounted on a Nikon inverted fluorescence microscope (Nikon Ti2-E). For the zebrafish experiments, a 10× objective lens (CFI Plan Apochromat Lambda D 10× 0.45 NA, Nikon) and a 20× objective lens (CFI Plan Apochromat Lambda D 20× 0.8 NA, Nikon) were used, while the tri-color experiments employed a 60× objective lens (CFI Apo TIRF 60× 1.49 NA, Nikon). The rest of the experiments were conducted using a 100× objective lens (CFI SR HP Apo TIRF 100× 1.49 NA, Nikon). A multi-mode laser (CELESTA Light Engine, Lumencor) served as the light source, paired with multi-band dichroic mirrors (CELESTA-DA/FI/TR/Cy5/Cy7-A-000, Semrock) for multi-color imaging (DAPI, FITC, TRITC, Cy5). The excitation laser was homogenized and transmitted to the illumination path via a multi-mode fiber (90-10719, 0.4 mm FC/PC, Lumencor), and collimated using a collimating lens (MAD406-A, *f* = 50.0 mm, LBTEK). The beam spot size was slightly larger than the DMD (DLP670S, Texas Instruments) target surface to fully utilize the excitation light energy. The beam was then modulated by the DMD to generate structured illumination, which passed through a pair of relay lenses (MAD510-A, *f* = 150 mm, LBTEK) before entering the microscope’s side port via the SD. The SD, with pinholes arranged in an Archimedean spiral pattern, was conjugated with both the DMD plane and the sample plane. It achieved stable, high-speed rotation at 5000 rpm using a brushless motor, and allowed for disk area switching via a motorized translation stage (MT1/M-Z9, Thorlabs). A 1:1 relay lens (105 mm F2.8 MACRO, SIGMA) was connected to the sCMOS camera (ORCA-Flash4.0 V3, Hamamatsu) to facilitate large FOV imaging. The main body of the microscope was equipped with a nano-positioning piezo sample scanner (NanoScan SP400, Prior) to achieve *z*-axis scanning.

### Instrument control

The microscope achieved synchronous control of all equipment through 3 analog signals and 2 digital signals output by the data acquisition card (USB-6363, NI) and LabVIEW (NI) programming. One analog signal was used for the external triggering of the DMD, another for the external triggering of the sCMOS, and a third for controlling the piezoelectric sample scanner. The DMD operated in Pattern On-The-Fly Mode, with the DMD exposure time synchronized with the camera’s exposure time, and the DMD dark time matched to the camera’s readout time. Two digital signals were used to achieve synchronization and arbitrary switching of the light sources. Two digital signals were used as addressing codes to cooperate with the DMD exposure time to achieve synchronization and arbitrary switching of the light sources through the demultiplexer. The image acquisition time sequence is illustrated in Fig. S3.

Setting the rotation speed *n* (rpm) and exposure time *t* (s) based on the SD’s spiral cluster number *N* enables the auto-synchronization between field scanning and image acquisition. As long as the angle rotated by the SD during the exposure time is an integer multiple of the angle required for a single uniform scan of the entire FOV, captured images are ensured to be exposed uniformly.

### Evaluation metrics

To assess the ability of the SD to remove out-of-focus signals, we evaluated the simulated images, multifocal images, and fringe images using Michelson contrast (MC), LC, FC, and CR.

MC is typically applied to patterns with equally bright and dark features that occupy similar proportions, calculated using the maximum grayscale value (${I}_{\max }$) and minimum grayscale value (${I}_{\min }$) of the image:

2 $${\rm{MC}}=({I}_{\max }-{I}_{\min })/({I}_{\max }+{I}_{\min })$$

LC quantifies the visibility of spots in multifocal images, calculated by the root mean square contrast of the sub-image *I* (size: *M* × *N*) containing a single spot:

3 $${\rm{LC}}=\sqrt{\mathop{\sum }\limits_{i=0}^{M-1}\mathop{\sum }\limits_{j=0}^{N-1}{({I}_{i,j}-\bar{I})}^{2}/MN}$$

FC measures the visibility of stripes in an image, determined by calculating the ratio of the intensities of the sidebands (*E*_sideband_) to the DC component (*E*_dc_) in the image’s frequency spectrum ($\tilde{I}$).

4 $$\begin{array}{c}{\rm{FC}}={E}_{{\rm{sideband}}}/({E}_{{\rm{dc}}}+{E}_{{\rm{sideband}}})\\ {E}_{{\rm{sideband}}}=\sum _{(u,v)\in^{\rm{sideband}}_{\rm{region}}}{|\tilde{I}(u,v)|}^{2}\,{E}_{{\rm{dc}}}={|\tilde{I}(0,0)|}^{2}\end{array}$$

CR measures the clarity of the sample imaging:

5 $${\rm{CR}}=\mathop{\sum}\limits_{\delta}\delta {(i,j)}^{2}{p}_{\delta }(i,j)$$

Where δ represents the difference in grayscale between adjacent pixels *i* and *j*, and *p*_δ_ denotes the probability distribution of pixels where the grayscale difference between adjacent pixels is δ.

We used PSMR and SSIM to evaluate the fidelity of super-resolution reconstruction results. The two metrics focus on pixel-level absolute differences and perceptual similarity, respectively.

6 $${\rm{MSE}}=\frac{1}{MN}\mathop{\sum }\limits_{i=1}^{M}\mathop{\sum }\limits_{j=1}^{N}{({X}_{i,j}+{Y}_{i.j})}^{2}$$

7 $${\rm{PSNR}}=10{\log}_{10}\left(\frac{{I}_{\max}^{2}}{MSE}\right)$$

8 $${\rm{SSIM}}=\frac{(2{\mu }_{X}{\mu }_{Y}+{C}_{1})(2{\sigma }_{XY}+{C}_{2})}{({{\mu }_{X}}^{2}+{{\mu }_{Y}}^{2}+{C}_{1})({{\sigma }_{X}}^{2}+{{\sigma }_{Y}}^{2}+{C}_{2})}$$

Where *X* and *Y* denote the two normalized images to compare, *μ* denotes the average value, *σ* denotes the covariance, *C*_1_ = 0.01, *C*_2_ = 0.03 a common empirical choices.

To evaluate the linear correlation between images, we randomly selected *N* points from the reference image (*I*_ref_) and then took the grayscale value at the same position from the result image degraded by RSF (*I*_convolved-res_). Set a hard threshold to exclude background points, and the linear correlation of the algorithm can be quantified by calculating the *R*² of the grayscale difference between the two images.

9 $$\Delta {I}_{{\rm{ref}}/{\rm{convolved}}-{\rm{res}}}={I}_{{\rm{ref}}/{\rm{convolved}}-{\rm{res}}}-\,\min ({I}_{{\rm{ref}}/{\rm{convolved}}-{\rm{res}}})$$

10 $${R}^{2}=1-\frac{{\sum }_{i=1}^{N}{({I}_{{\rm{convolved}}-{\rm{res}}}^{(i)}-{I}_{{\rm{ref}}}^{(i)})}^{2}}{{\sum }_{i=1}^{N}{({I}_{{\rm{convolved}}-{\rm{res}}}^{(i)}-{\bar{I}}_{{\rm{convolved}}-{\rm{res}}})}^{2}}$$

We performed NanoJ-SQUIRREL analysis to estimate the artifacts in the reconstructed (https://github.com/superresolusian/NanoJ-SQUIRREL), which includes the calculation of RSE and RSP, as well as the generation of super-resolution images after RSF degradation and the associated error map.

FRC and image decorrelation were used to evaluate the resolution of the reconstruction results quantitatively, which are open-sourced at https://github.com/sakoho81/miplib and https://github.com/Ades91/ImDecorr.

### Deconvolution algorithm

The data reconstructed through DPA-PR can be further enhanced in resolution through deconvolution. The data presented in the manuscript were processed using Huygens (SVI, Netherlands) with default parameters.

### Sample preparation

#### Fixed sample

The fixed sample used for tri-color imaging (Mitochondria, F-actin, and nuclei) in Fig. 5a was purchased from ThermoFisher (FluoCells #1, F36924). The mouse kidney section in Figs. 1g, 2b–c, and 5c–e was purchased from ThermoFisher (FluoCells #3, F24630). The sample of Aspergillus conidiophores in Figs. 6b–e was purchased from Carolina Biological Supply (297872, American). Kidney section sample in Fig. 4h–l was purchased from Suzhou Shenying Optical Co., Ltd.

## Zebrafish culture and preparation

The zebrafish (three days post-fertilization) were anesthetized with Tricaine, and placed in a confocal dish. Then the zebrafish was embedded into a 1.5% low-melting-point agarose to make it as close as possible to the coverslip. At the same time, adjust the posture so that the head is downward, and wait for the agarose gel to solidify before imaging.

## Supplementary information

Supporting Information for “High-fidelity tissue super-resolution imaging achieved with confocal^2^ spinning-disk image scanning microscopy”

Supplementary Movie S1. The Effect of Spinning Disk on DMD Masks

Supplementary Movie S2. The Process of Spinning Disk Scanning Across Full Field of View

Supplementary Movie S3. Reconstruction Process of the DPA-PR Algorithum

Supplementary Movie S4. The Three-Dimensional, Large-Field-of-View Imaging Result of C^2^SD-ISM in Zebrafish

Supplementary Movie S5. The Three-Dimensional Imaging Result of the Mold Sample

## Data Availability

The data that support the findings of this study are available from the corresponding author upon reasonable request.

## Code availability

The reconstruction algorithms, simulation scripts, and hardware control codes associated with this work are available at https://github.com/Chauncey-Leung/C2SD-ISM.
