# Supplementary material for: High-fidelity tissue super-resolution imaging achieved with confocal2 spinning-disk image scanning microscopy
Source: Light Sci Appl. 2025 Aug 4;14:260. doi: 10.1038/s41377-025-01930-x (PMC12319088; doi:10.1038/s41377-025-01930-x)
Supplement: Supplementary file 1 — Supporting Information for “High-fidelity tissue super-resolution imaging achieved with confocal2 spinning-disk image scanning microscopy” [file 41377_2025_1930_MOESM1_ESM.pdf]

Supporting Information

High-fidelity tissue super-resolution imaging achieved with confocal^2 spinning-disk image scanning microscopy

Qianxi Liang[1, †], Wei Ren[1, †], Boya Jin[2], Liang Qiao[2], Xichuan Ge[2] , Yunzhe Fu[1], Xiaoqi Lv[3], Meiqi Li[3, *], Peng Xi[1, *]

[1] Department of Biomedical Engineering, National Biomedical Imaging Center, College of Future Technology, Peking University, Beijing 100871, China

[2] Airy Technologies Co. Ltd., Beijing 100086, China

[3] School of Life Sciences, Peking University, Beijing 100871, China

[†] Equal Contribution.

*Correspondence should be addressed to P.X. (xipeng@pku.edu.cn) or M.L. (limeiqi@pku.edu.cn).

Table of content:

Fig. S1 to S15...03

Note S1. Image formation model...21

Note S2. Optimization of the DMD incident angle......................................24

Note S3. The Design for spinning disk..27

Note S4. Detailed process of DPA-PR reconstruction...................................30

Note S5. Simulation of the effect of SD on DMD masks C^2SD-ISM imaging.........33

Note S6. Comparison of ISM techniques based on digital reconstruction...............35

Table S1. Comparison of ISM techniques based on digital reconstruction...........36

References...37

Other supporting materials for this manuscript include the following:

Movie S1. The Effect of SD on DMD Masks.

Movie S2. The Process of Spinning Disk Scanning Across Full Field of View.

Movie S3. Reconstruction Process of the DPA-PR Algorithum.

Movie S4. The Three-Dimensional, Large-Field-of-View Imaging Result of C^2SD-ISM in Zebrafish.

Movie S5. The Three-Dimensional Imaging Result of the Mold Sample.

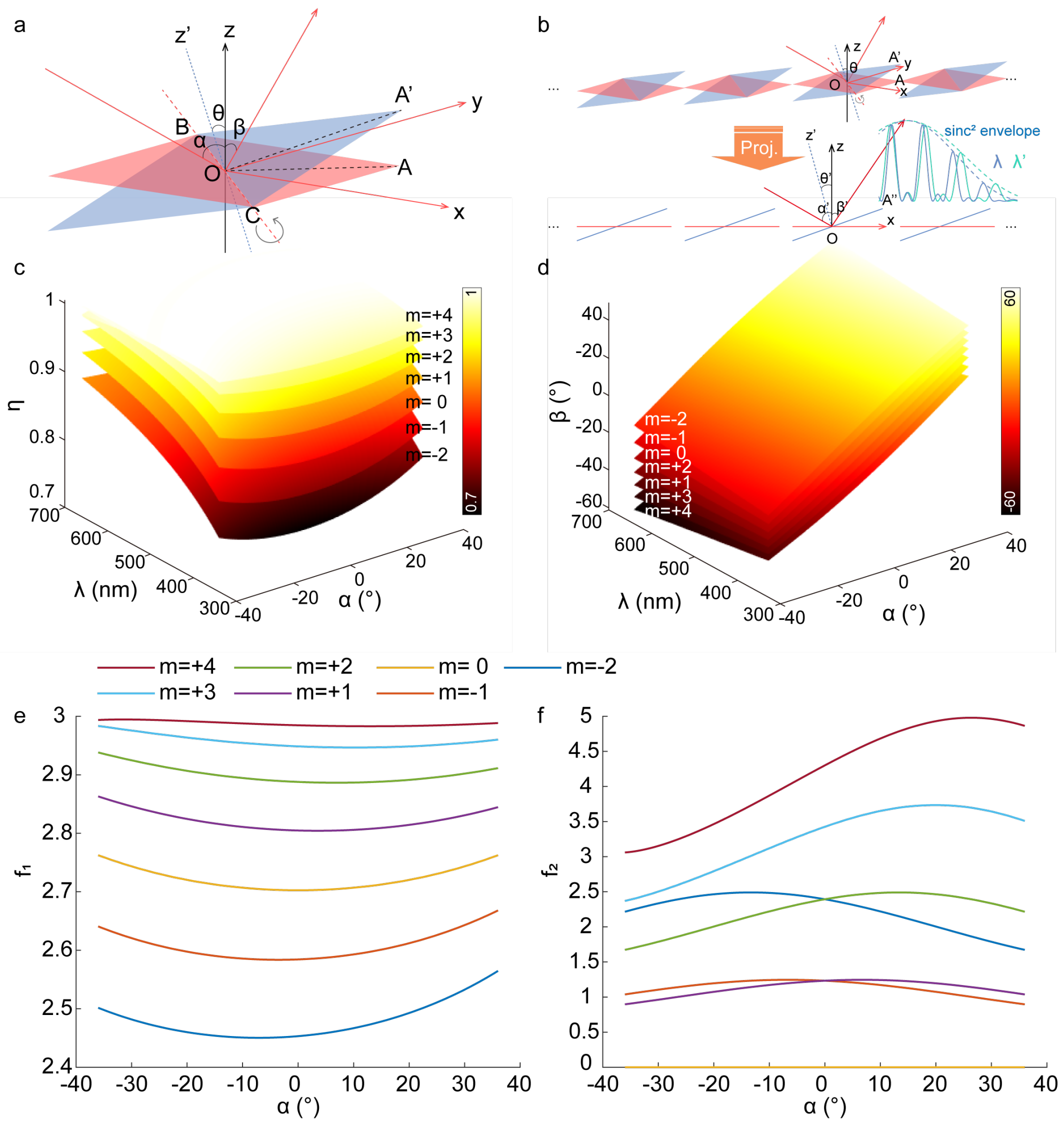

Fig. S1. Schematic diagram of the DMD geometry and simulation results of its diffraction properties. **a** Spatial geometry of a single micromirror in the flat state and ON state. The red plane represents the flat state of the micromirror, and the blue plane represents the ON state, with an angle of θ between them. The black solid line Oz is the normal of the red plane, and the blue dashed line Oz' is the normal of the blue plane. Points A and A' are the same vertex of the micromirror. The red dashed line BC denotes the rotation axis. The red solid lines represent the x-axis, y-axis, incident light, and exit light, all of which pass through the center point O. The x-axis and y-axis lie within the red plane and are parallel to the edges of the flat-state micromirror. The incident and exit light lie in the plane AOA' and form angles of α and β with Oz, respectively. **b** Diagram of a series of micromirrors arranged in one direction and their projection in plane xOz. The angles α', β', and θ' are the projections of the spatial angles α, β, and θ, respectively, with the remaining symbols having the same meanings as in Fig S1a. **c**

Quantitative relationship between diffraction efficiency η, wavelength λ, and incident angle α. **d** Quantitative relationship between exit angle β, wavelength λ, and incident angle α. **e** Variation of loss function f_1 under different incident angles α and diffraction orders m. **f** Variation of loss function f_2 under different incident angles α and diffraction orders m.

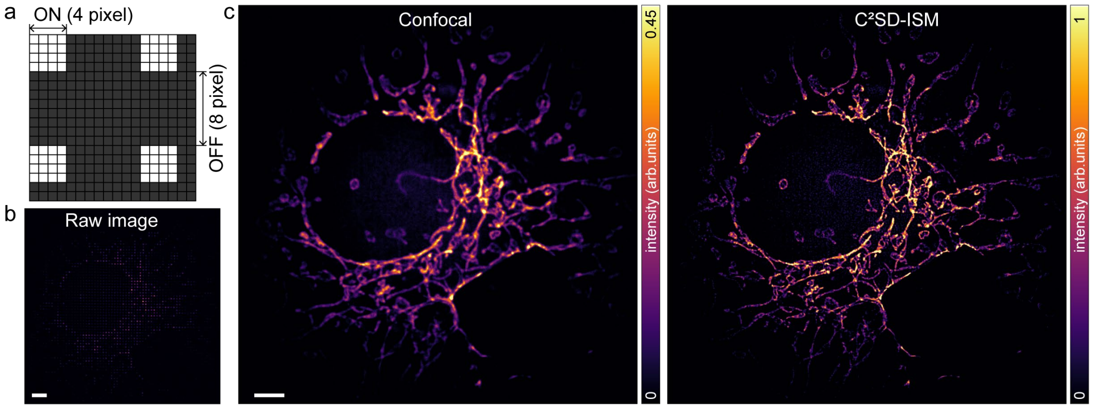

Fig. S2. **a** Schematic of the DMD mask applied during 36-frame acquisition. **b** The raw image under 36-frame acquisition. **c** Confocal and C^2SD-ISM imaging results of mitochondria using 36 frames for reconstruction. Scale bars: 5 µm.

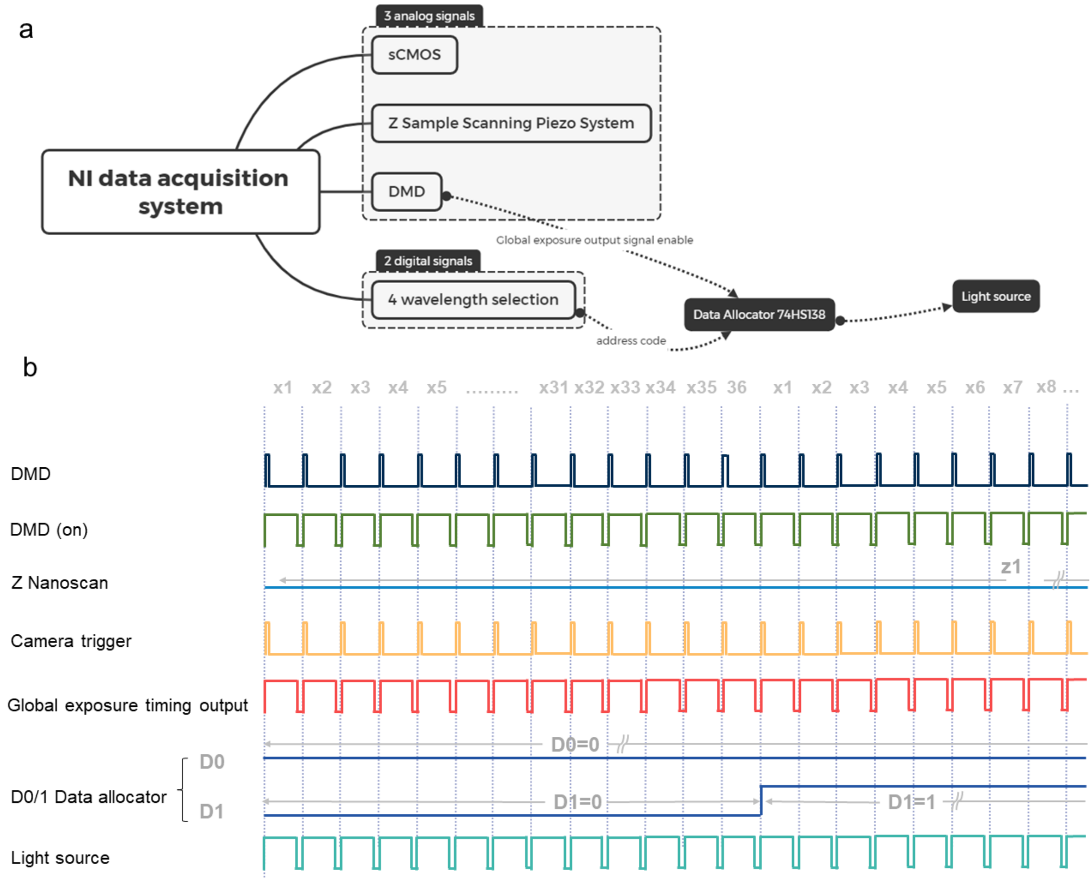

Fig. S3. Instrument control and data acquisition process of C^{2}SD-ISM. **a** Overview of the instrument control. **b** Data acquisition process for multi-*z* and multi-color imaging. See Methods “Instrument control” for a detailed description.

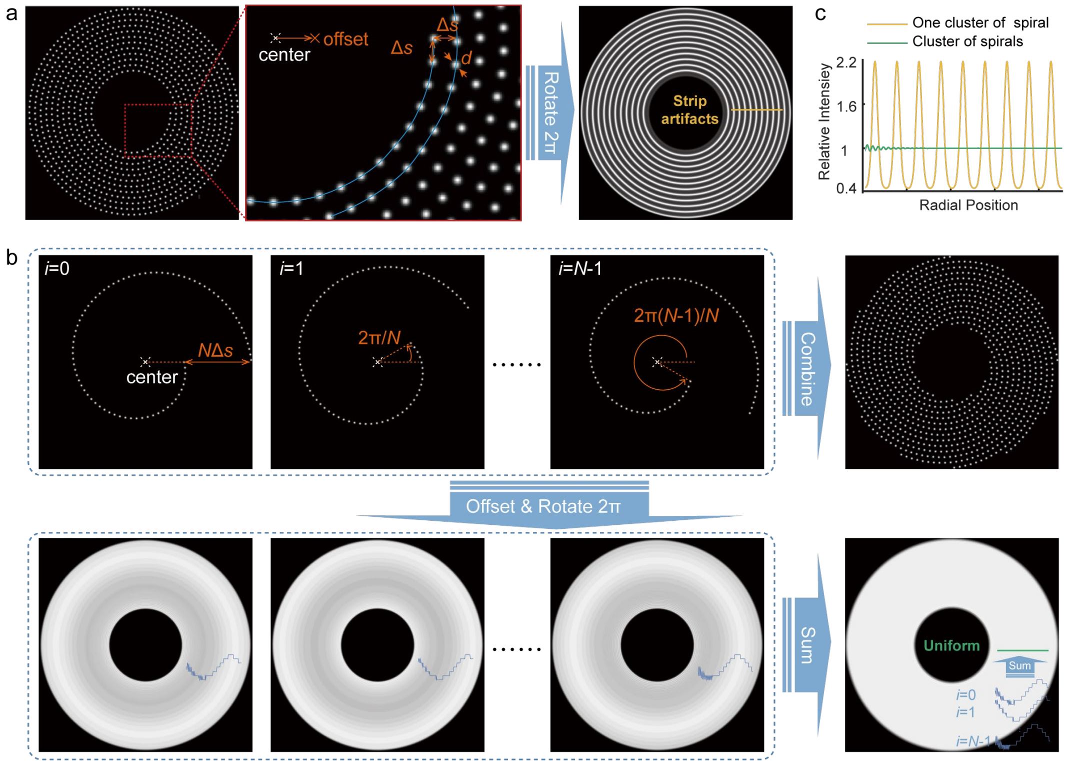

Fig. S4. Schematic diagram of large N resistance to the centering offset error. **a** Schematic diagram of spinning disk configuration parameters and anti-center error. Pinhole arrangement on the SD follows an Archimedean spiral with a constant radial step Δs, and a pinhole diameter d; the positional deviation between the motor's rotational center and the pinhole pattern center is indicated as offset. The center offset is set to 500 μm. **b** Diagram showing the effect that increasing N can improve the system's robustness to center aligning requirement. **c** Intensity distribution of FOV illumination for one and multi cluster Archimedean spirals configuration (here N is 1 or 12). Values are taken from the yellow and green lines in Fig S4a and Fig S4b.

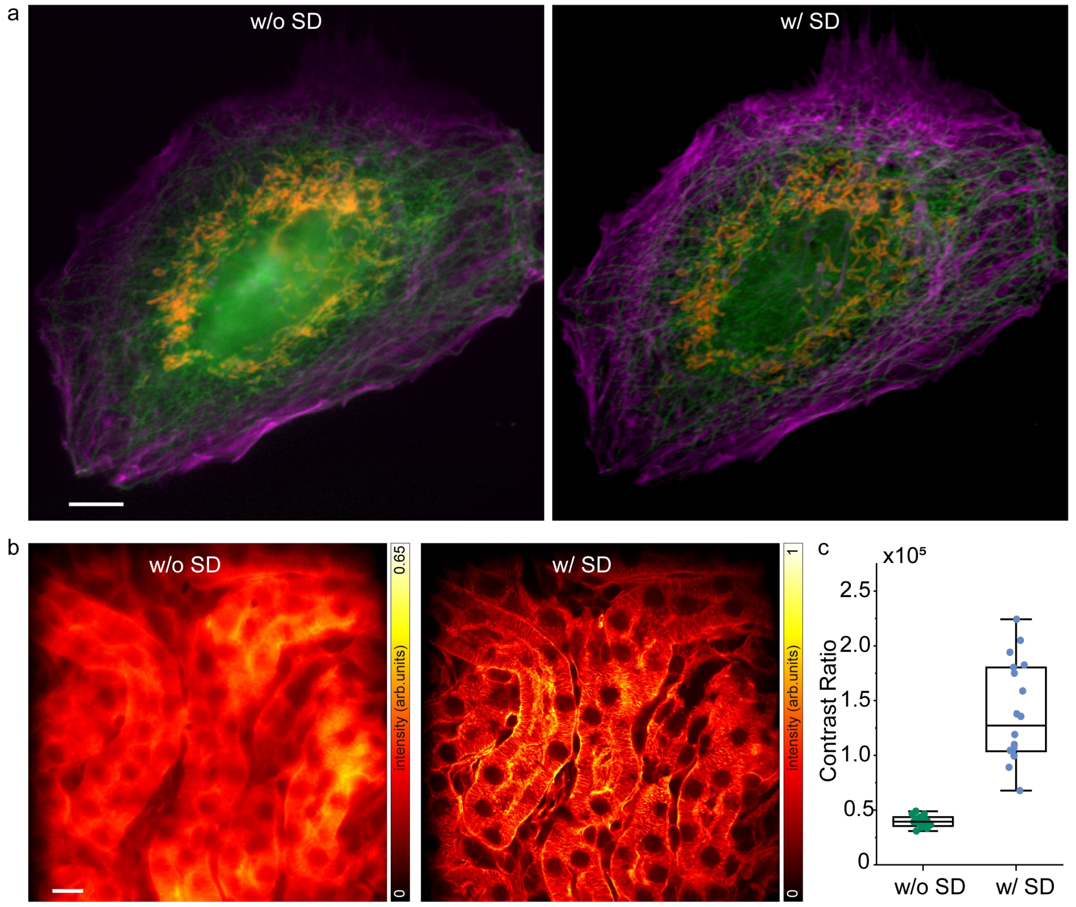

Fig. S5. Comparison of imaging results with and without SD setup. **a**Tri-color imaging of U2OS cells. The 488 channel labels microtubules using antibodies with FITC as the secondary antibody; the 561 channel labels mitochondria using an RFP plasmid; and the 640 channel labels actin using Phalloidin-637 dye. Scale bars: 7 µm. **b** The 16 µm cryostat section of mouse kidney stained with Alexa Fluor 488 wheat germ agglutinin. Scale bar: 10 µm. **c** Box plot of contrast ratio measurements.

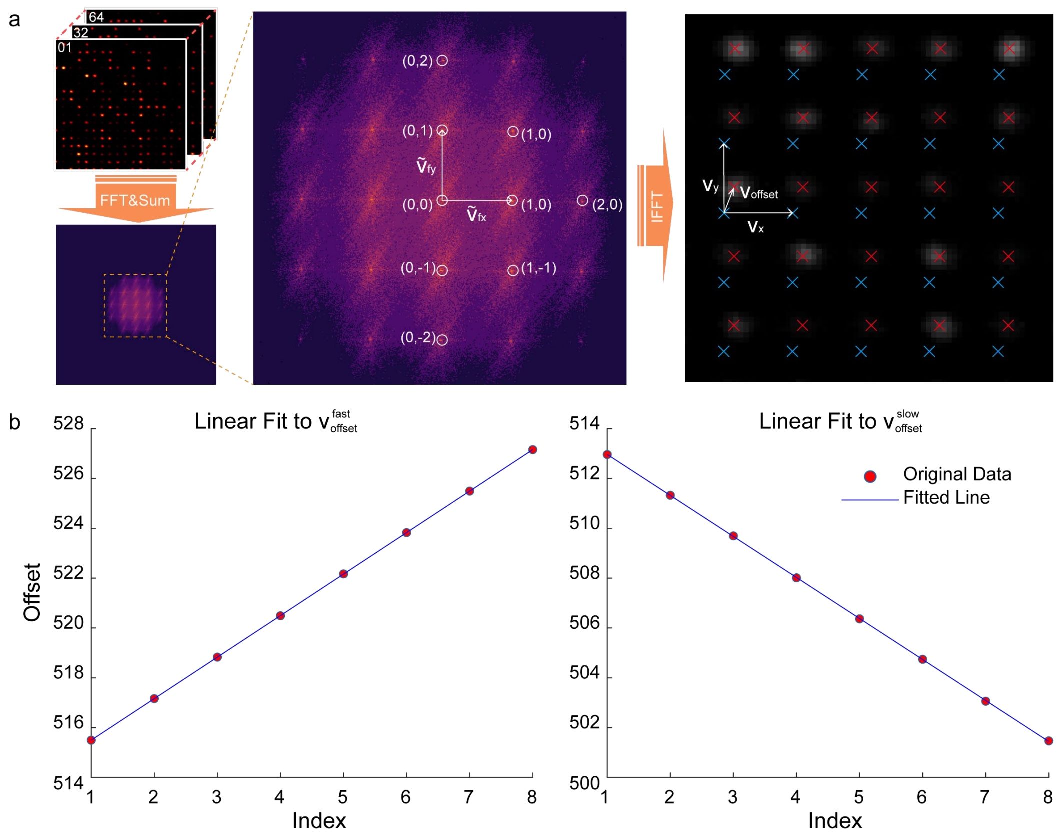

Fig. S6. Schematic diagram of excitation optical axis array localization and VDA pitch determination. **a** Process of excitation optical axis array localization. The middle panel shows the summed Fourier spectrum of the raw frames, exhibiting a lattice distribution. The red circles mark the lattice points of the spectrum, with $\tilde{\mathbf{v}}_{fx}$ and $\tilde{\mathbf{v}}_{fy}$ as the basis vectors of the spectrum lattice. The right panel shows the raw frame, where $\tilde{\mathbf{v}}_{fx}$ and $\tilde{\mathbf{v}}_{fy}$ are transformed back into the spatial domain via inverse Fourier transform to obtain $\mathbf{v}_x$ and $\mathbf{v}_y$. The blue crosses represent the lattice generated by $\mathbf{v}_x$ and $\mathbf{v}_y$, and the red crosses indicate the final lattice positions corrected by $\mathbf{v}_{\text{offset}}$. **b** Linear fitting of the stepping distances of $\mathbf{v}_{\text{offset}}$ to determine the lattice stepping distances $\mathbf{v}_{\text{offset}}^{\text{fast}}$ and $\mathbf{v}_{\text{offset}}^{\text{slow}}$, which are then set as the VDA pitch.

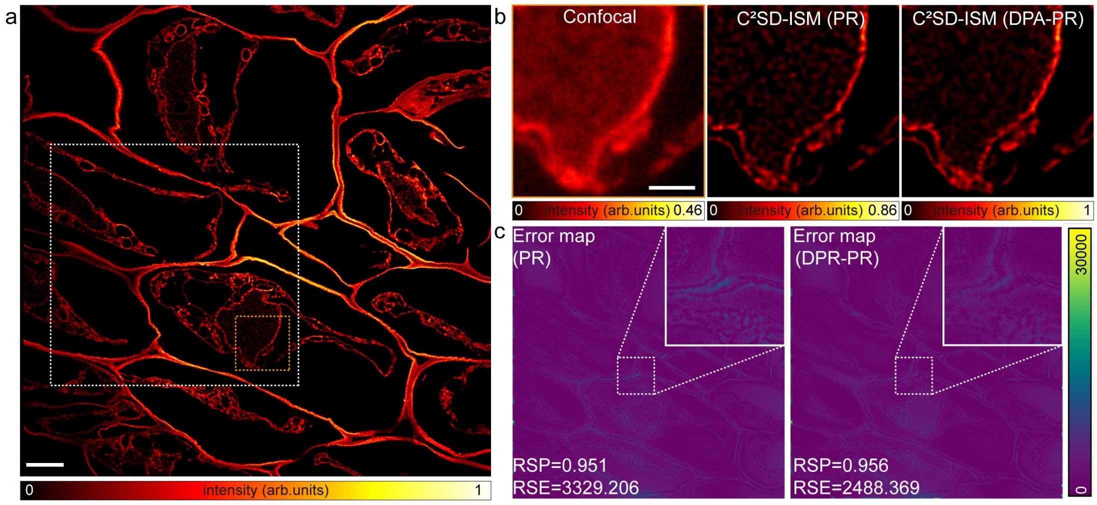

Fig. S7. **a** Imaging results of a kidney section sample using C^{2}SD-ISM. Scale bar: 5 μm. The white dashed box indicates the cropped region used in Fig 2b and Fig 2c. **b** Enlarged view of the orange dashed box region in Fig S7a, showing a comparison of confocal, PR, and DPA-PR. **c** Error maps of super-resolution reconstruction results under PR and DPA-PR, generated by NanoJ-SQUIRREL with the confocal image as the reference. Scale bar: 2 μm.

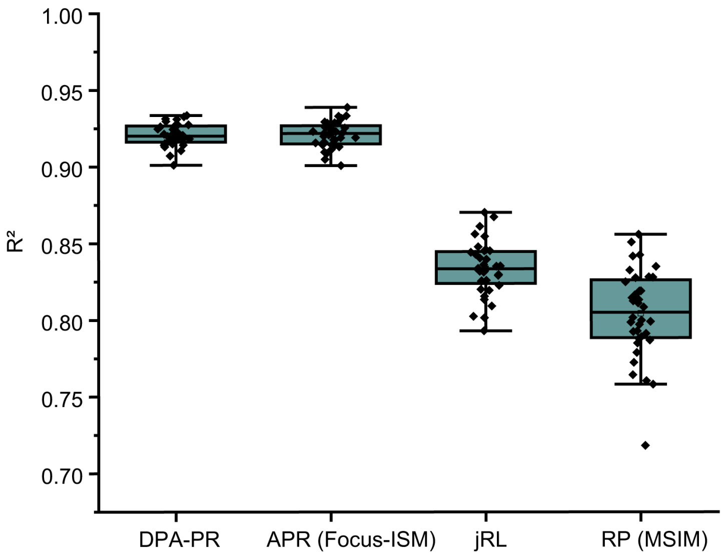

Fig. S8. Comparison of the linear correlation of DPA-PR with APR[1], jRL[2] and MSIM-PR[3].

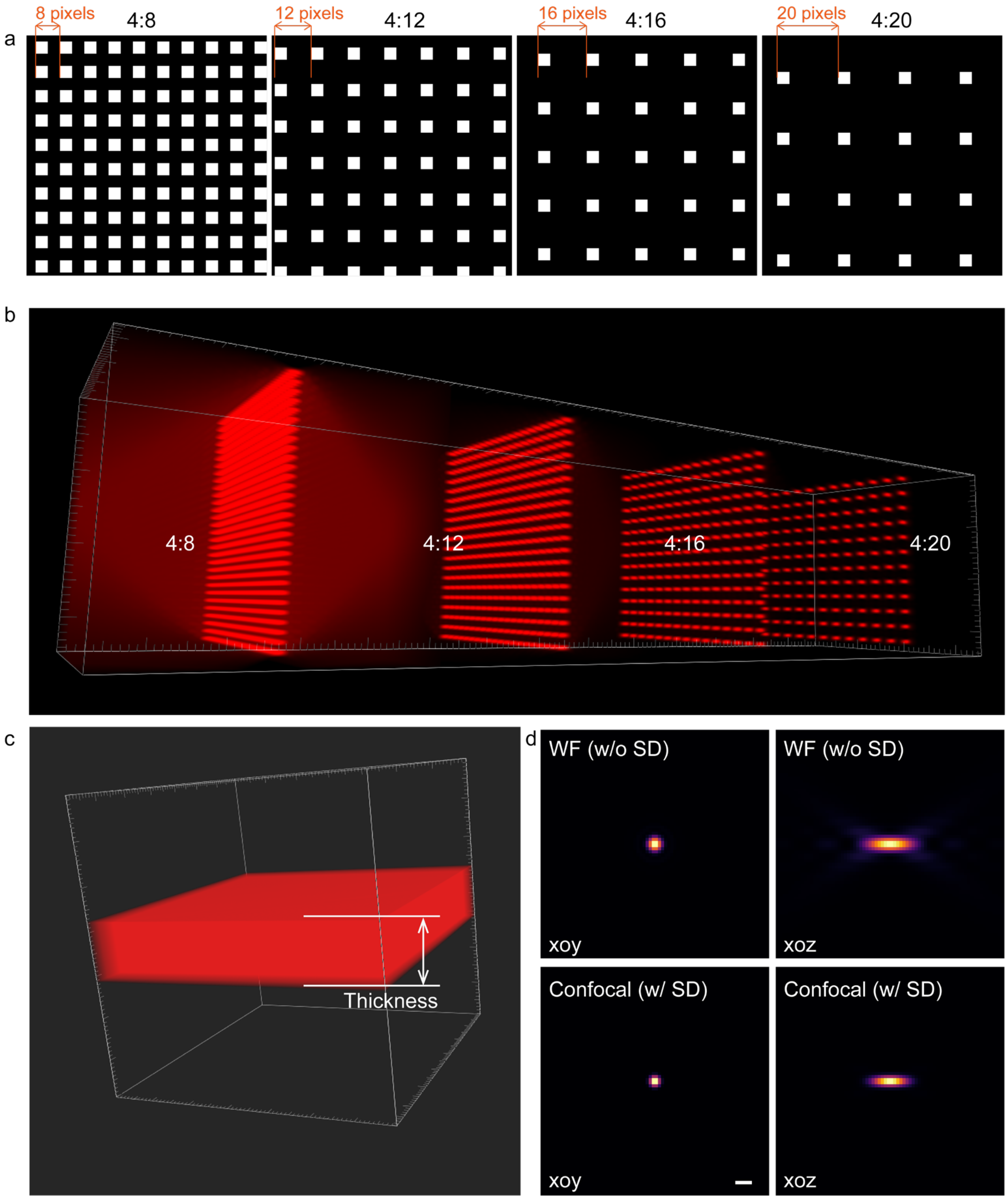

Fig. S9. Simulation of the effect of SD on the DMD mask. **a** Schematic of DMD masks with different spacings. **b** Illumination patterns formed by different DMD masks. **c** Sample structure used in the simulation. **d** PSF used in the simulation. Scale bar: 1 μm.

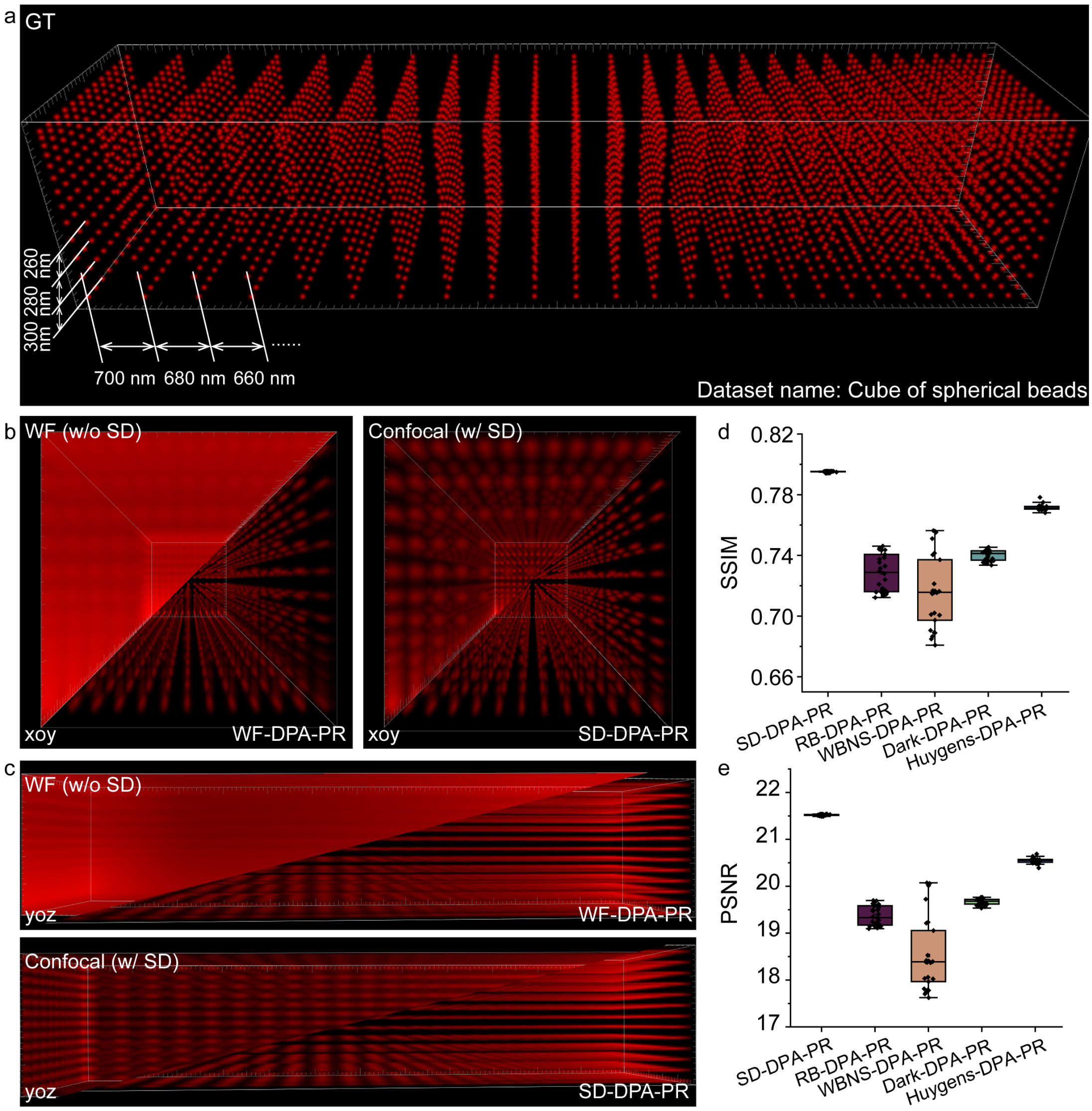

Fig. S10. Simulation results using the cube of spherical beads as the 3D synthetic dataset. **a** Schematic of the cube of spherical beads as the GT for simulation. **b** 3D views of imaging results along the *xoy* plane under different configurations. **c** 3D views of imaging results along the *yoz* plane under different configurations. **(d, e)** Quantitative comparison of super-resolution results and GT images using SSIM and PSNR metrics.

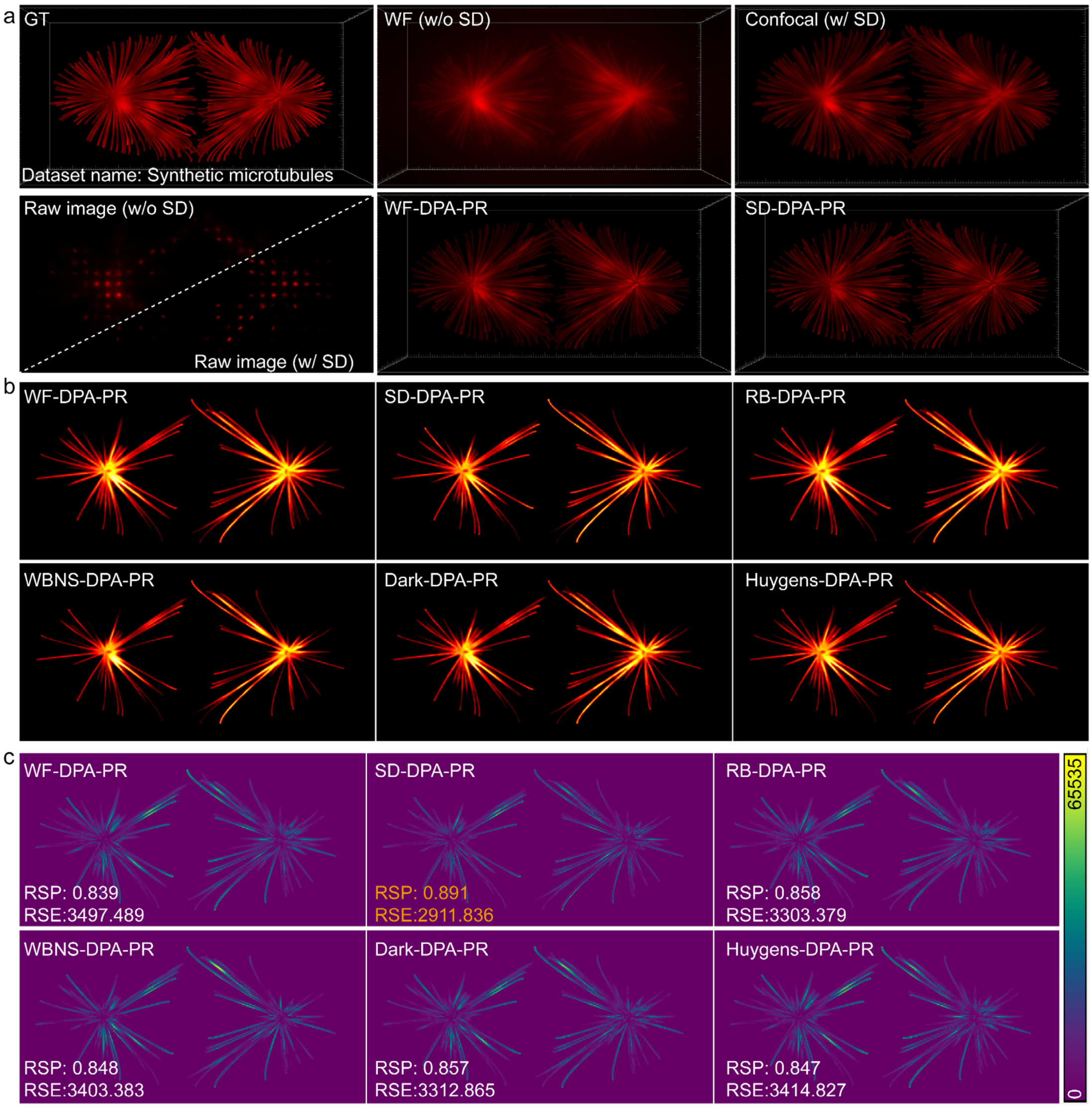

Fig. S11. Simulation results using microtubules as the 3D synthetic dataset. **a** 3D views of ground truth (GT), wide-field imaging (w/o SD), confocal imaging (w/ SD), DPA-PR reconstruction without the SD (WF-DPA-PR), and DPA-PR reconstruction with the SD (SD-DPA-PR). The bottom-right inset compares a single raw frame acquired under multi-focus illumination with and without the SD. **b** The super-resolution results include DPA-PR reconstruction without the SD (WF-DPA-PR), DPA-PR reconstruction with the SD (SD-DPA-PR), and DPA-PR reconstruction without the SD but preprocessed using different background removal algorithms (RB-DPA-PR, WBNS-DPA-PR, Dark-DPA-PR and Huygens-DPA-PR). **c** Error maps of super-resolution reconstruction results under different conditions, generated by NanoJ-SQUIRREL with the GT image as the reference. The results show that the SD-DPA-PR reconstruction is the closest to the GT image, with the best RSP and RSE metrics.

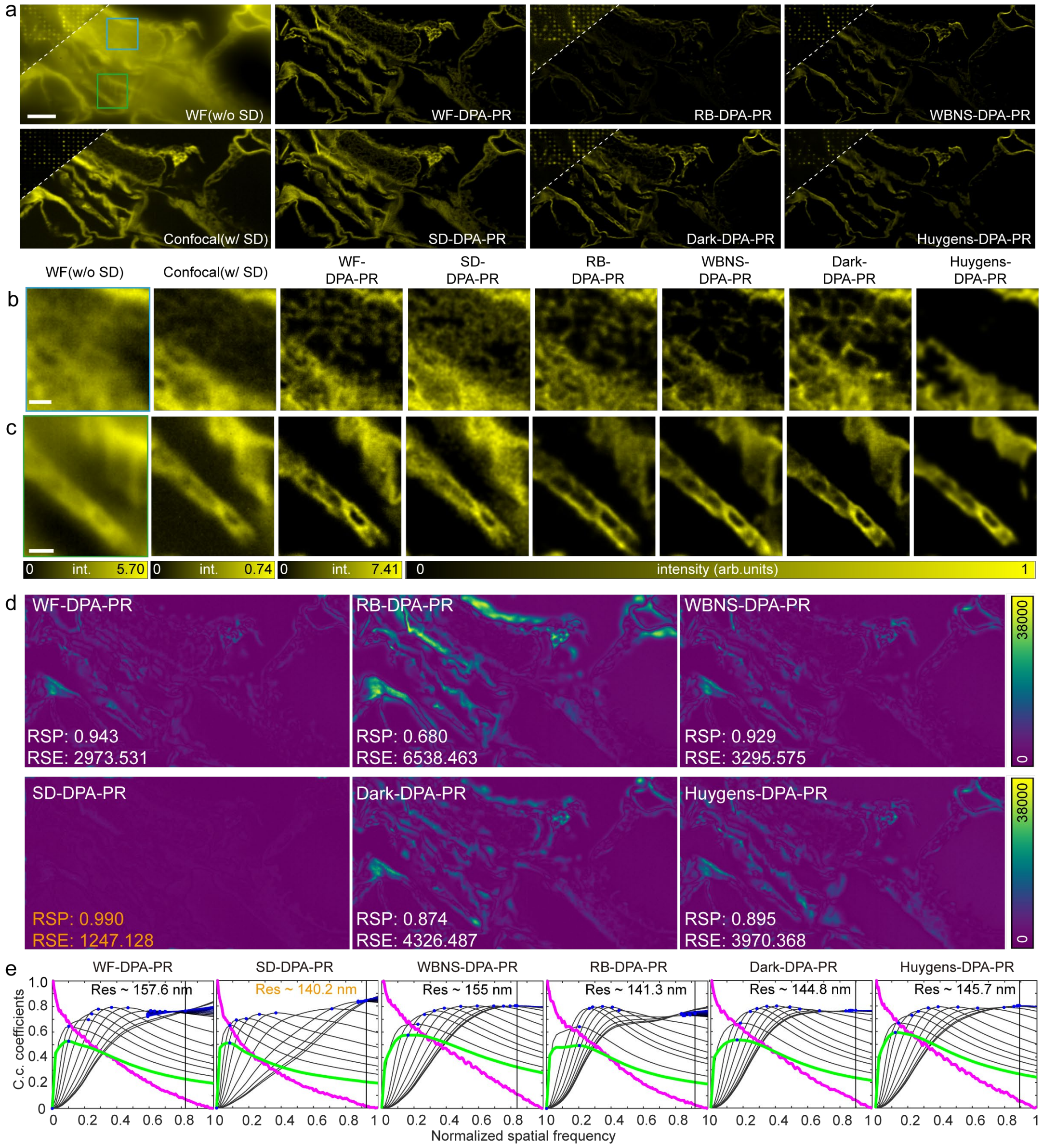

Fig. S12. Effect of the SD on C^2SD-ISM imaging of a 12 μm thick mouse kidney section sample. **a** Imaging results include wide-field (w/o SD), confocal (w/ SD), DPA-PR reconstruction without the SD (WF-DPA-PR), DPA-PR reconstruction with the SD (SD-DPA-PR), and DPA-PR reconstruction without the SD but preprocessed using different background removal algorithms (RB-DPA-PR, WBNS-DPA-PR, Dark-DPA-PR and Huygens-DPA-PR). **(b, c)** Magnified views of the regions indicated by the green and blue rectangles in Fig S12a, respectively. **d** Error maps of super-resolution reconstruction results under different conditions, generated by NanoJ-SQUIRREL with the confocal image as the reference. The analysis demonstrated that the SD-DPA-PR reconstruction achieved the best RSP and RSE metrics. **e** Quantitative resolution assessment using image decorrelation analysis, revealing that all DPA-PR results

surpassed the diffraction limit, and SD-DPA-PR achieved the highest resolution. Scale bar: 5 μm in Fig S12a; 1 μm in Fig S12b and Fig S12c.

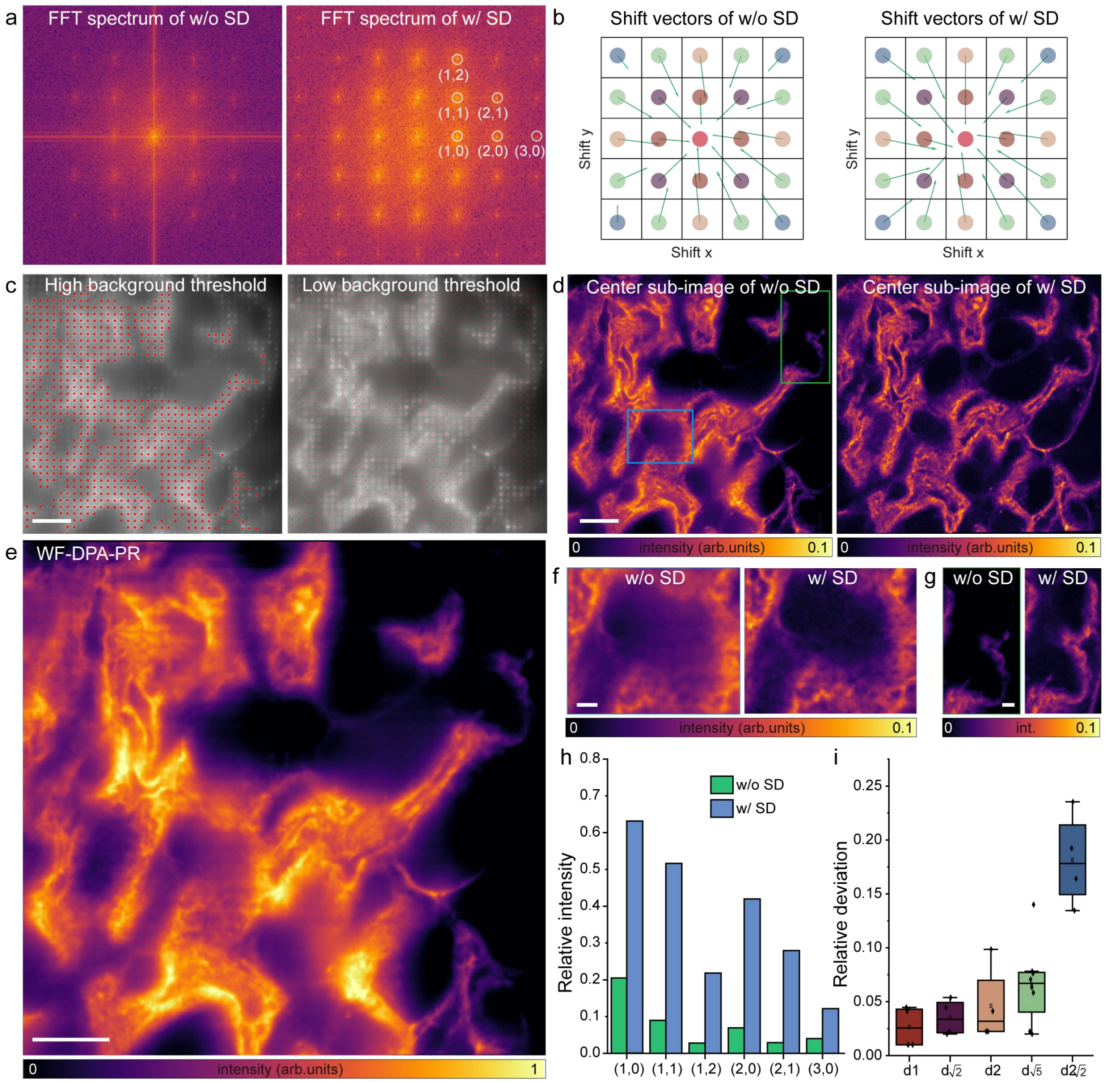

Fig. S13. Failure of direct super-resolution reconstruction on wide-field images of thick tissues. **a** Comparison of Fourier spectrum of raw images without (left) and with (right) the SD. The order point intensities are weaker without SD. **b** Comparison of estimated shift vectors without and with the SD. **c** Points positioning of the raw frames and background thresholding setting without the SD. High background thresholds lead to excessive background inclusion, while low thresholds discard much in-focus information. **d** Comparison of extracted central sub-images with and without the SD. **e** WF-DPA-PR results, filled with defocus signals and artifacts in high-signal regions, with signal loss at structural edges. **f** Magnified view of the blue box in Fig S13d, showing artifacts due to excessive background inclusion without the SD. **g** Magnified view of the green box in Fig S13d, showing structural loss due to in-focus signals being filtered as background without the SD. **h** Bar chart of relative intensities of different orders in a compared to the central DC region. The introduction of the SD significantly

increases the relative intensity of diffraction orders, with the (3,0) order exceeding the (1,1) order without the SD. **i** Relative deviation of the shift vector calculated without the SD compared to with the SD. The box color represents the sub-image's distance from the axis, corresponding to the VDA element colors in Fig S13b. The further the sub-image is from the center, the larger the shift vector deviation. Scale bars: 10 μm in Fig S13c, Fig S13d, and Fig S13e; 2 μm in Fig S13f and Fig S13g.

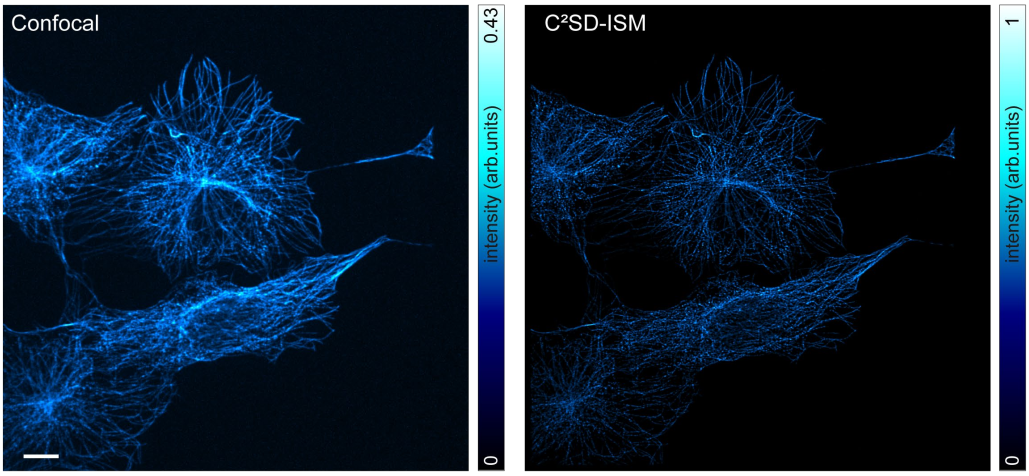

Fig. S14. Comparison of confocal imaging and C^{2}SD-ISM super-resolution imaging of microtubule in fixed cells. Scale bar: 5 μm.

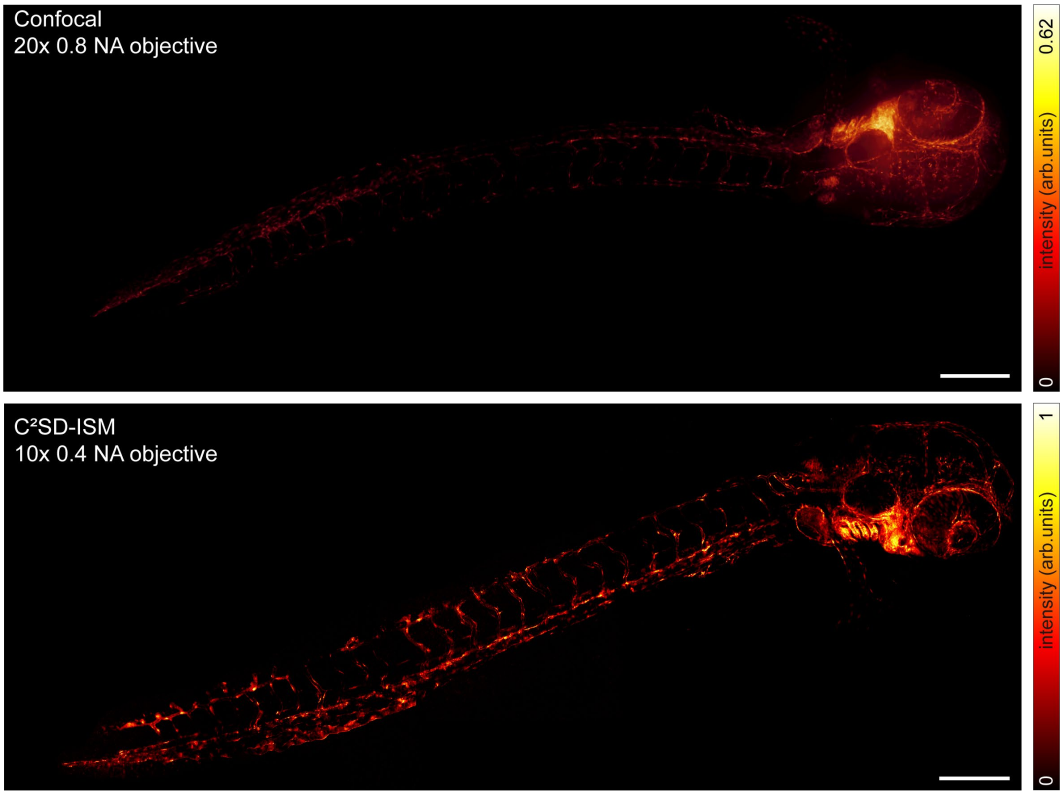

Fig. S15. Comparison of confocal imaging and C^{2}SD-ISM super-resolution imaging in zebrafish under different objectives configuration. The top image shows confocal imaging results of a zebrafish acquired using a 20× 0.8 NA objective lens, while the bottom image shows C^{2}SD-ISM super-resolution imaging results of another zebrafish acquired using a 10× 0.4 NA objective lens. Scale bar: 200 μm.

Note S1. Image formation model

The key feature of a confocal imaging system is the detection pinhole, which is conjugated to the excitation pinhole, effectively blocks out-of-focus signals. The point detector positioned behind the detection pinhole captures signals exclusively from the object's focal plane. By moving the excitation and detection pinholes synchronously, the beam can scan different positions on the object. Let the plane coordinates of the excitation pinhole, the object's focal plane, and the detection pinhole be denoted as $\mathbf{r}_1$, ξ and $\mathbf{r}_2$, respectively. Since the absolute position of the sample within the focal plane and on the CCD does not need to be considered, a unit magnification is assumed for simplicity. Consequently, the light intensity I detected by the detector at different positions $\mathbf{r}$ on the sample is given by:

$$\begin{aligned} I(\mathbf{r}) &= \int \mathrm{d}\mathbf{r}_2 A_{\mathrm{det}}(\mathbf{r}-\mathbf{r}_2) \int d\xi \mathrm{PSF}_{\mathrm{det}}(\mathbf{r}_2-\xi) O(\xi) \int \mathrm{d}\mathbf{r}_1 A_{\mathrm{ex}}(\mathbf{r}_1-\mathbf{r}) \mathrm{PSF}_{\mathrm{ex}}(\xi-\mathbf{r}_1) \\ &= \left\{\left[\mathrm{PSF}_{\mathrm{ex}}(-\mathbf{r}) \otimes A_{\mathrm{ex}}(-\mathbf{r})\right] \cdot \left[\mathrm{PSF}_{\mathrm{det}}(\mathbf{r}) \otimes A_{\mathrm{det}}(\mathbf{r})\right]\right\} \otimes O(\mathbf{r}) \end{aligned} \quad \text{(S1)}$$

Where $\mathrm{PSF}_{\mathrm{ex}}$ and $\mathrm{PSF}_{\mathrm{det}}$ represent the excitation and detection PSF, respectively. O denotes the sample distribution. A_{ex} and A_{det} denote the excitation pinhole and the detection pinhole, respectively.

We used a DMD to modulate the excitation light in the excitation optical path, enabling structured illumination for the object. Focusing on the excitation process, this can be modeled as the switching modulation of the excitation pinhole. Since the DMD plane is conjugate to the object's focal plane, let the pattern loaded onto the DMD be denoted as M. If the scanned position corresponds to a "OFF" pixel (or region) on the DMD, no light is transmitted, and the light intensity is zero, effectively closing the pinhole. Conversely, if the corresponding DMD pixel (or region) is "ON", it is equivalent to opening the pinhole. Therefore, the imaging formation model for C^2SD - ISM is:

$$I(\mathbf{r}) = \left\{\left[\mathrm{PSF}_{\mathrm{ex}}(-\mathbf{r}) \otimes \left(A_{\mathrm{ex}}(-\mathbf{r}) M(-\mathbf{r})\right)\right] \cdot \left[\mathrm{PSF}_{\mathrm{det}}(\mathbf{r}) \otimes A_{\mathrm{det}}(\mathbf{r})\right]\right\} \otimes O(\mathbf{r}). \quad \text{(S2)}$$

Assuming the excitation pinhole is infinitely small, it can be modeled as a Dirac delta function:

$$A_{\mathrm{ex}}(\mathbf{r}) = \delta(\mathbf{r}). \quad \text{(S3)}$$

Consequently, the imaging model simplifies to the following form:

$$I(\mathbf{r}) = m\left\{\mathrm{PSF}_{\mathrm{ex}}(-\mathbf{r})\cdot\left[\mathrm{PSF}_{\mathrm{det}}(\mathbf{r})\otimes A_{\mathrm{det}}(\mathbf{r})\right]\right\}\otimes O(\mathbf{r}) \quad m = M\,|_{r=0}\,. \tag{S4}$$

In the C[2]SD-ISM system, the DMD is employed to implement structured illumination by sequentially loading a set of masks onto it, which includes $\{M_1, M_2, \ldots, M_N\}$. As the DMD cycles through these masks, a corresponding set of raw images is recorded, which we can represent as $\{I_1, I_2, \ldots, I_N\}$. The total number of raw images is N. For a specific image sample position $\mathbf{r}_0$, we have:

$$I_i(\mathbf{r}_0) = m_i I(\mathbf{r}_0) \quad i = 1,2\ldots,N. \tag{S5}$$

When imaging thicker samples, we applied the method of zero-frequency removal technique along the modulation direction to eliminate stray signals, further suppressing the background and removing the camera's offset gray value[4]. As demonstrated by the Fourier transform properties, the zero-frequency signal represents the average value of the original sequence. Therefore, the image after such preprocessing becomes:

$$\tilde{I}_i(\mathbf{r}_0) = m_i I_i(\mathbf{r}_0) - \overline{m} I_i(\mathbf{r}_0) \quad \overline{m} = \sum_{i=1}^{N} m_i / N. \tag{S6}$$

$$\Delta\tilde{I}_i(\mathbf{r}_0) = (m_i - \overline{m})\Delta I_i(\mathbf{r}_0) \tag{S7}$$

It is evident that $\Delta\tilde{I}_i(\mathbf{r}_0)$ exhibits a linear relationship with $\Delta I_i(\mathbf{r}_0)$, preserving the relative intensities before and after preprocessing. In some cases, the value of $m_i - \overline{m}$ may be less than zero, indicating that the fluorescence intensity is below the average level. In such case, we interpret this as being primarily due to pinhole crosstalk. Even though it may contain minimal sample information, its contribution to the sample reconstruction is negligible, and thus, we set it to zero.

ISM employs a detection array, where each pixel can be considered equivalent to a point detector with an offset detection pinhole. From (S1), it can be observed that if the pinhole offset is $\mathbf{r}_n$, the PSF of the system becomes:

$$\mathrm{PSF}_{\mathrm{sys}}(\mathbf{r};\mathbf{r}_n) = \mathrm{PSF}_{\mathrm{ex}}(-\mathbf{r})\cdot\left[\mathrm{PSF}_{\mathrm{det}}(\mathbf{r}-\mathbf{r}_n)\otimes A_{\mathrm{det}}(\mathbf{r})\right]. \tag{S8}$$

The OTF of the system can be obtained by applying the Fourier transform to (S8):

$$\mathrm{OTF}_{\mathrm{sys}}(\mathbf{f};\mathbf{r}_n) = \mathrm{OTF}_{\mathrm{ex}}(-\mathbf{f})\otimes\left[\exp(-2\pi\mathbf{f}\mathbf{r}_n)\mathrm{OTF}_{\mathrm{det}}(\mathbf{f})\mathcal{A}_{\mathrm{det}}(\mathbf{f})\right]. \tag{S9}$$

There is also a spatial shift in the acquired image, assuming that the shift is $s\mathbf{r}_n$ (the method for obtaining s will be introduced later, which differs significantly between traditional PR and DPA-PR). Compensating for this shift and stacking all shifted images results in the effective OTF of ISM:

$$\mathrm{OTF_{ISM}}(\mathbf{f};\mathbf{r}_n)=\int \mathrm{OTF_{sys}}(\mathbf{f};\mathbf{r}_n)\exp(i2\pi s\mathbf{f}\mathbf{r}_n)\mathrm{d}\mathbf{r}_n\,. \tag{S10}$$

The existence of an explicit $\mathrm{OTF_{ISM}}$ expression shows that ISM reconstruction is also a linear process.

In conventional PR, assuming both $\mathrm{PSF_{ex}}$ and $\mathrm{PSF_{det}}$ follow Gaussian distributions:

$$h_{\mathrm{exc}}(\mathbf{r};0)\propto \exp\left\{-\frac{\mathbf{r}^2}{2\sigma_{\mathrm{exc}}^2}\right\},\quad h_{\mathrm{det}}(\mathbf{r};\mathbf{r}_n)\propto \exp\left\{-\frac{(\mathbf{r}-\mathbf{r}_n)^2}{2\sigma_{\mathrm{det}}^2}\right\}. \tag{S11}$$

Their product results in a new Gaussian function, whose center is offset from the optical axis. This offset **p** corresponds to the required shift $s\mathbf{r}_n$ for correcting the signal back to its appropriate spatial location:

$$\mathbf{p}(\mathbf{r}_n)=s\mathbf{r}_n=\frac{\sigma_{\mathrm{exc}}^2}{\sigma_{\mathrm{exc}}^2+\sigma_{\mathrm{det}}^2}\cdot\mathbf{r}_n\,. \tag{S12}$$

Assuming $\sigma_{\mathrm{exc}}=\sigma_{\mathrm{det}}$, the shift becomes:

$$\mathbf{p}(\mathbf{r}_n)=\frac{1}{2}\mathbf{r}_n,\quad s=\frac{1}{2}. \tag{S13}$$

This conventional PR is intuitive and simple. However, it has limitations. In real fluorescence microscopy, excitation and emission wavelengths differ due to the Stokes shift (i.e. $\sigma_{\mathrm{exc}}\neq\sigma_{\mathrm{det}}$). Moreover, the ideal PSF in a diffraction-limited system is an Airy pattern, given by: $2\left(J_1(v)/v\right)^2$ $v=2\pi\mathrm{NA}r/\lambda$, which deviates from the Gaussian approximation. In the presence of optical aberrations or other non-idealities, this deviation becomes more pronounced, potentially leading to inaccurate shift estimation using the traditional PR model.

The DPA-PR method we proposed can adaptively estimates the shift vector **p** via normalized cross-correlation between the confocal sub-image detected by the edge detector $i(\mathbf{r};\mathbf{r}_n)$ and that detected by the central detector $i(\mathbf{r};0)$:

$$\mathbf{p}(\mathbf{r}_n)=\underset{\mathbf{r}}{\mathrm{argmax}}\,\mathcal{F}^{-1}\left\{\frac{\mathcal{F}\{i(\mathbf{r};\mathbf{r}_n)\}\mathcal{F}^*\{i(\mathbf{r};0)\}}{\left|\mathcal{F}\{i(\mathbf{r};\mathbf{r}_n)\}\mathcal{F}^*\{i(\mathbf{r};0)\}\right|}\right\}, \tag{S14}$$

where $\mathcal{F}$ and $\mathcal{F}^{-1}$ denote the Fourier and inverse Fourier transforms. the superscript asterisk * denotes the complex conjugate. By relying on actual recorded data, DPA-PR adapts to realistic PSF distortions and spectral differences, making it more consistent with the physical principles of ISM and more robust under experimental conditions. Detailed reconstruction procedures of the DPA-PR method are provided in Note S4.

Note S2. Optimization of the DMD incident angle

The DMD is an array of micromirrors that exhibits the diffraction properties of a 2D blazed grating. Previous studies in the field of Structured Illumination Microscopy (SIM) have utilized DMD as a diffraction element to generate stripe-patterned structured illumination[5,6]. However, to ensure high diffraction efficiency and equal ±1-order diffraction light intensity, the incident and the exit angle must satisfy the blazing conditions. In C^2SD-ISM system, DMD functions as a projection device. Light of different wavelengths is incident at the same angle, but the diffracted light will have an angular offset and a shift in diffraction efficiency. These offsets need to be within the allowable range of the imaging scene.

As shown in the Fig S1a, a single micromirror in the flat state is represented by the red plane (in the plane ABC), and the x- and y-axes (Ox||AB, Oy||AC) are aligned along the edges of the micromirror. The normal to the center O of the micromirror is the z-axis. In the ON state, the micromirror rotates by an angle θ around the diagonal BC, tilting to the blue plane (in the plane A′BC, A rotates to A′). The normal to the blue plane is Az', then ∠zOz′=θ. For the convenience of optical alignment and manipulation, we positioned the optical axis in the plane AOA′, then the incident angle α and the exit angle β are the angles between the incident beam and the exit beam and Oz. Both angles lie in the plane AOA′. When the glare condition is satisfied,

$$\beta = \alpha - 2\theta. \tag{S15}$$

To study the DMD as a 1D diffraction grating, we projected the plane AOA′ onto the plane xOz. α, β and θ on the plane AOA′ map to α′, β′ and θ′ on the plane xOz, respectively (Fig S1b). For simplicity, assume the side length of the micromirror be 2, then the coordinates of point A are (1,1,0). let ∠AOA′=γ, the coordinates of point A′ are $\left(\cos\gamma/\sqrt{2}, \cos\gamma/\sqrt{2}, \sin\gamma\right)$. The point A is projected to point A″ on the plane xOz, and ∠AOA′ after being projected onto the plane xOz is ∠AOA″=γ′.

Using spatial analytic geometry, line OA:

$$\frac{\sqrt{2}x}{\cos\gamma} = \frac{\sqrt{2}y}{\cos\gamma} = \frac{z}{\sin\gamma}. \tag{S16}$$

Line OA″:

$$\begin{cases} \dfrac{\sqrt{2}x}{\cos\gamma} = \dfrac{z}{\sin\gamma} \\ y = 0 \end{cases} \Rightarrow \begin{cases} z = \left(\sqrt{2}\tan\gamma\right)x \\ y = 0 \end{cases}. \tag{S17}$$

Thus, the relationship between γ and γ′ can be derived:

$$\tan\gamma' = \sqrt{2}\tan\gamma \Rightarrow \gamma' = \arctan\left(\sqrt{2}\tan\gamma\right). \quad \text{(S18)}$$

Next, angle θ′ can be obtained by letting γ=θ+π/2:

$$\tan\left(\theta' + \pi/2\right) = \sqrt{2}\tan\left(\theta + \pi/2\right) \Rightarrow \theta' = \arctan\left(\tan\theta/\sqrt{2}\right). \quad \text{(S19)}$$

Similarly,

$$\beta' = \arctan\left(\tan\beta/\sqrt{2}\right) \quad \alpha' = \arctan\left(\tan\alpha/\sqrt{2}\right). \quad \text{(S20)}$$

The exit angle needs to be the direction of the diffraction main maximum, as follows:

$$d\left(\sin\alpha' + \sin\beta'\right) = m\lambda \quad m = 0, \pm1, \pm2, ... \quad \text{(S21)}$$

$$\beta'\left(\alpha;\lambda\right) = \arcsin\left(m\lambda/d - \sin\alpha'\right) \quad \text{(S22)}$$

$$\beta\left(\alpha;\lambda\right) = \arctan\left(\sqrt{2}\tan\left(\beta'\right)\right). \quad \text{(S23)}$$

Where d is the period of the micromirror array. The corresponding diffraction efficiency η is:

$$\eta\left(\alpha;\lambda\right) = \left\{\frac{\sin\left[\pi a\left(\sin\left(\alpha' - \theta'\right) - \sin\left(\beta' + \theta'\right)\right)/\lambda\right]}{\pi a\left(\sin\left(\alpha' - \theta'\right) - \sin\left(\beta' + \theta'\right)\right)/\lambda}\right\}^2. \quad \text{(S24)}$$

Where α is the gap between two adjacent micromirrors, whose value can be estimated according to $a = d\left(1 - \sqrt{s}\right)$, where s is the array filling factor of DMD. The DMD used is d=5.4 μm, s=0.93, which can simulate the quantitative relationship between diffraction efficiency, exit angle, wavelength and incident angle (Fig S1c, S1d).

To demonstrate multicolor imaging, we considered a tri-color mitochondrial sample, with three excitation channels at λ_1=561 nm, λ_2=488 nm, and λ_3=405 nm. The goal is to optimize the incident angle such that the diffraction efficiency for all three channels is maximized and the difference in exit angles is minimized. To quantify this, two evaluation functions, f_1 and f_2, are defined:

$$f_1\left(\alpha\right) = \sum_{i=1}^{3}\eta\left(\alpha;\lambda_i\right) \quad \text{(S25)}$$

$$f_2\left(\alpha\right) = \max\left(\left|\beta\left(\alpha;\lambda_1\right) - \beta\left(\alpha;\lambda_2\right)\right|, \left|\beta\left(\alpha;\lambda_2\right) - \beta\left(\alpha;\lambda_3\right)\right|\right). \quad \text{(S26)}$$

Where f_1 reflects the total diffraction efficiency of the three channels, and f_2 represents the maximum deviation of the exit angle between the wavelengths on the longest and shortest from the middle wavelength. As shown in Fig S1e and S1f, higher diffraction

orders can ensure high diffraction efficiency, but lead to a larger difference in exit angles between wavelengths. Fortunately, even when m=4, the maximum deviation of the exit angle is only 4.9° (corresponding to an incident angle of 26.3°). The deviation of the illumination region on the sample plane is completely acceptable (at the cost reduced FOV at the edges). In this case, the diffraction efficiency for all three channels exceeds 0.95. For this incident angle of 26.3°, the corresponding exit angle are:

$$\beta\left(26.3^{\circ};\lambda_1\right)=-6.9^{\circ}\quad \beta\left(26.3^{\circ};\lambda_2\right)=-2.5^{\circ}\quad \beta\left(26.3^{\circ};\lambda_3\right)=2.5^{\circ}. \tag{S27}$$

These exit angles are nearly collinear, with a small deviation around 0°, making them ideal for optical alignment and device arrangement.

Note S3. The Design for spinning disk

The arrangement of pinholes on the surface of the SD involves distributing them at different radial and angular positions along a spiral. Typically, the pinholes are arranged in one or more clusters of Archimedean spirals. A polar coordinate system is established with the center of the SD as the pole. The equation for the Archimedean spiral is given by:

$$\rho = \rho_{\text{start}} + \frac{N\Delta s}{2\pi}\left(\theta - \frac{2\pi}{N}i\right) \quad i = 0,1,...,N-1. \tag{S28}$$

Where ρ and θ represent the radial and angular positions of the point in the polar coordinate system, N is the number of concentric Archimedean spiral clusters. When N=1, (S28) simplifies to a single cluster Archimedean spiral equation. The index i ranges from 0 to $N-1$, representing different Archimedean spirals, with the only difference between them being the angular offset $2\pi/N$. The value ρ_{start} represents the radial position of the starting point of the pinhole's distribution, and the spiral pitch is denoted by Δs. We set the pinhole spacing, i.e., the arc length of the Archimedean spiral between adjacent pinholes along the Archimedean spiral, equal to the spiral pitch.

It is observed that due to the constant angular velocity of the SD, the larger the radial position of the pinholes, the greater the linear velocity. Let Φ_e represent the irradiance of the light incident on the SD, ω be the angular velocity of the SD, and r be the radius of the pinhole. For an area element $\rho_0\delta\rho\delta\theta$ located at (ρ_0, θ_0), the exposure in the angular direction is proportional to the time the pinhole passes through the area (which is inversely proportional to the linear velocity of the pinhole). The exposure in the radial direction is proportional to the number of times the pinhole passes through the area (which is inversely proportional to the radial sampling rate s_ρ). Therefore, the radiant energy for this area element is:

$$Q_e \propto \Phi_e \pi r^2 \frac{s_\rho}{\omega\rho_0}. \tag{S29}$$

Taking the difference of (S29) gives:

$$\delta\rho = \frac{N\Delta s}{2\pi}\delta\theta. \tag{S30}$$

According to $\delta\theta \approx \Delta s / \rho$, the sampling rate can be expressed as:

$$s_\rho = \frac{1}{\delta\rho} = \frac{2\pi\rho}{N\Delta s^2} \tag{S31}$$

Inserting $s_\rho|_{\rho=\rho_0}$ into (S29) we can get

$$Q_e \propto 2\pi^2 \frac{\Phi_e r^2}{N\omega\Delta s^2}. \tag{S32}$$

It can be observed that Q_e is independent of the location of pinholes, meaning that this pinholes distribution ensures uniform illumination across different areas when the SD rotates.

The pinholes on the SD are fabricated by etching, which may introduce centering errors. As shown in Fig S4c, the larger the N, the more robust the system is to the center aligning requirement. When the deviation is 50 μm, obvious artifacts appear in the FOV due to uneven illumination when N=1, whereas the FOV remains largely unaffected when N=12.

The principle is illustrated in Fig S4. The offset is set as 500 μm. A cluster of N=12 spirals can be divided into separate sub-spirals. After each sub-spiral completes a 2π rotation, it covers the entire FOV more comprehensively than a single spiral (N=1), although it does not achieve perfectly uniform scanning on its own. Notably, the bright and dark areas created by the rotation of sub-spirals with different i values are staggered, and the superposition of complementary gray values creates a uniformly illuminated FOV as shown by the blue lines, leading to a uniform illumination in the entire field of view.

However, N is not necessarily better when larger. The radial increment between adjacent pinholes must satisfy the Nyquist sampling theorem. According to (S31), increasing N or decreasing ρ will increase the radial stepping distance $\delta\rho$, Therefore, when designing the SD, the outer area of the SD should be utilized as much as possible, and the number of spiral clusters N should not be too large.

In addition to the design of the disk pattern, the values of specific parameters also need to be considered. The first is the size of pinholes. In confocal, both the excitation and detection pinhole diameters are usually 1 AU. For a 100× objective with a 1.49 NA, the pinhole diameter is usually around 50 μm. To balance the light power and defocusing effect, the pinhole spacing is usually 5 times the pinhole diameter, i.e., 250 μm, resulting in a filling factor of 0.031. The ratio of the diameter to spacing of the pinhole is independent of the objective lens, allowing the SD to be compatible with objectives of any magnification. The pinhole diameter and spacing can be adjusted based on the imaging requirements. If greater defocusing tolerance is needed, the

pinhole spacing can be increased, or the diameter can be reduced. Conversely, if higher signal transmittance is required, the pinhole diameter can be increased, or the spacing can be reduced. To accommodate different imaging needs, a single SD can be partitioned into sections with different radial ranges, with each section having a distinct diameter-to-spacing ratio.

Note S4. Detailed process of DPA-PR reconstruction

The process of DPA-PR reconstruction includes the following main steps: (1) Excitation optical axis array localization; (2) VDA sampling; and (3) Registration and superposition.

(1) Excitation optical axis array localization: The location of the excitation optics axis is the center of the illumination spot. However, the position of the maximum gray value in the acquired illumination spot image may not correspond to the actual excitation optics axis due to modulation of the excitation spot by the sample structure. Based on the prior knowledge that the excitation optical spot follows a square lattice pattern, we can obtain the basis vector $\mathbf{v}_x$ and $\mathbf{v}_y$ (ideally, $||\mathbf{v}_x||=||\mathbf{v}_y||$). By generating lattice points using these vectors and calculating the average deviation between the generated lattice points and the brightest points of the illumination, the offset vector $\mathbf{v}_{\text{offset}}$ can be obtained. The vectors $\mathbf{v}_x$, $\mathbf{v}_y$ and $\mathbf{v}_{\text{offset}}$ can be used to determine the exact location of the excitation optical axis array. The specific process is as follows:

a. Background removal preprocessing. A Fourier transform is performed pixel by pixel on the 3D image stack along the scanning direction. Since the background stray signal is relatively uniform at a macroscopic scale and does not change with the movement of excitation spots, it exhibits a direct current characteristic. The background of the acquired images is further removed by eliminating the zero-frequency component.

b. Determine $\mathbf{v}_x$ and $\mathbf{v}_y$. Because the Fourier spectrum of the lattice in spatial domain is still a lattice, and the translation of the lattice in spatial domain does not change the corresponding positions in frequency domain, a Fourier transform is performed on the original images and superimpose their frequency spectra. Let the basis vectors in the spectrum lattice be $\tilde{\mathbf{v}}_{fx}$ and $\tilde{\mathbf{v}}_{fy}$, then the coordinates of the lattice points (marked by white circles in Fig S6a) are:

$$\begin{bmatrix} \tilde{x} \\ \tilde{y} \end{bmatrix} = \begin{bmatrix} \tilde{\mathbf{v}}_{fx} & \tilde{\mathbf{v}}_{fy} \end{bmatrix} \begin{bmatrix} m \\ n \end{bmatrix} \quad m,n = 0,\pm1,\pm2,... \tag{S33}$$

By locating the local maximum, coordinates of a series of lattice points $\begin{bmatrix} \tilde{\mathbf{x}} \\ \tilde{\mathbf{y}} \end{bmatrix} = \begin{bmatrix} \tilde{x}_{(0,1)} & \tilde{x}_{(1,0)} & \tilde{x}_{(1,1)} & \cdots \\ \tilde{y}_{(0,1)} & \tilde{y}_{(1,0)} & \tilde{y}_{(1,1)} & \cdots \end{bmatrix}$, and their indices $\begin{bmatrix} \mathbf{m} \\ \mathbf{n} \end{bmatrix} = \begin{bmatrix} 0 & 1 & 1 & \cdots \\ 1 & 0 & 1 & \cdots \end{bmatrix}$ can be determined, then $\tilde{\mathbf{v}}_{fx}$ and $\tilde{\mathbf{v}}_{fy}$ can be obtained by the least squares method:

$$\begin{bmatrix} \tilde{\mathbf{v}}_{fx} & \tilde{\mathbf{v}}_{fy} \end{bmatrix} = \begin{bmatrix} \tilde{\mathbf{x}} \\ \tilde{\mathbf{y}} \end{bmatrix} \begin{bmatrix} \mathbf{m} \\ \mathbf{n} \end{bmatrix}^T \left(\begin{bmatrix} \mathbf{m} \\ \mathbf{n} \end{bmatrix} \begin{bmatrix} \mathbf{m} \\ \mathbf{n} \end{bmatrix}^T \right)^{-1} . \quad \text{(S34)}$$

Transforming them back to the spatial domain gives us $\mathbf{v}_x$ and $\mathbf{v}_y$.

b. Determine $\mathbf{v}_{\text{offset}}$.

Use $\mathbf{v}_x$ and $\mathbf{v}_y$ to generate a lattice $\left\{\mathcal{L} \mid \mathcal{L}_i = (x_i, y_i)^T\right\}$ (marked by blue crosses in Fig S6a). The point spacing of this lattice and the excitation optical axis array should ideally be equal, but there is a deviation between their spatial position, which is described by $\mathbf{v}_{\text{offset}}$. We used the spatial maximum point of the spot $(x_i^m, y_i^m)^T$ as a reference, calculate the distance to the nearest lattice point as d_i, and average all d_i to eliminate the influence of the sample modulation on the excitation illumination spot. Each frame has its own $\mathbf{v}_{\text{offset}}$. We used $\mathbf{v}_{\text{offset}}^k$ to represent the $\mathbf{v}_{\text{offset}}$ of the k-th frame.

(2) VDA sampling: We assumed a 5×5 VDA, with its center located at the excitation optical axis, and moving synchronously with the excitation lattice scanning. The pitch between adjacent VDA pixels is equal to the scanning step size of the excitation lattice. The image is resampled by the VDA to obtain the pixel values detected at the scanning position of 25 sub-images. The key steps are as follows.

a. Determine the pitch of VDA. Taking 8×8 scanning as an example, the pattern loaded into the DMD is scanned line by line in a withdrawn manner. The pitch value is calculated based on $\mathbf{v}_{\text{offset}}^k$ (k=1, 2,…, 64). First, like phase unwrapping, the $\mathbf{v}_{\text{offset}}^k$ vector may differ from each other by a basic vector, so the series of $\mathbf{v}_{\text{offset}}^k$ need to be adjusted. Then, the 8×8 $\mathbf{v}_{\text{offset}}^k$ cell is averaged in the row and column directions and only take the x component and y component respectively to obtain $\mathbf{v}_{\text{offset}}^{\text{fast}}$ and $\mathbf{v}_{\text{offset}}^{\text{slow}}$. The increments of $\mathbf{v}_{\text{offset}}^{\text{fast}}$ and $\mathbf{v}_{\text{offset}}^{\text{slow}}$ is just the pitch of VDA and can be obtained through linear regression.

$$\text{pitch}_{\text{fast}}, \sim = \arg\min_{k,b} \left\| k\mathbf{x} + b - \mathbf{v}_{\text{offset}}^{\text{fast}} \right\|_2^2 \quad \mathbf{x} = [1, 2, ..., 8]^T , \quad \text{(S35)}$$

$$\text{pitch}_{\text{slow}}, \sim = \arg\min_{k,b} \left\| k\mathbf{x} + b - \mathbf{v}_{\text{offset}}^{\text{slow}} \right\|_2^2 \quad \mathbf{x} = [1, 2, ..., 8]^T . \quad \text{(S36)}$$

b. Determine gray values sampled by VDA.

The VDA pixel's pitch is not equal to the camera's pixel size. Assuming that the image grayscale value distribution follows the discrete function $I(\mathbf{r})$, where $\mathbf{r}$ represents the discrete camera pixel coordinates, we constructed the VDA pixel coordinates $\mathbf{r}'$ relative to $\mathbf{r}$. The values of $I(\mathbf{r}')$ were obtained by interpolating the raw camera image

$I(\mathbf{r})$. Specifically, under the imaging conditions of a 100× objective and a 1024×1024 camera field of view, a single image may contain over 5,000 illumination spots. For a 36-frame acquisition, this results in more than 100,000 interpolation operations. Although high-order interpolation methods such as bicubic or spline interpolation offer greater accuracy, they introduce substantial computational overhead. Conversely, nearest-neighbor interpolation is computationally efficient but lacks the spatial accuracy required. To achieve a balance between computational efficiency and interpolation precision, bilinear interpolation was employed to estimate the intensity values at the VDA positions.

(3) Registration and superposition: After 5×5 VDA sampling, 25 confocal sub-images are obtained. The reconstruction result is then obtained by aligning and superimposing the sub-images.

a. Registration. First, the image is apodised by multiplying a Hann window, and convolved with a Gaussian kernel to remove the noise. Then, phase cross-correlation (PCC)[7] is performed between the k-th edge sub-image I_k and center sub-image I_{center} (i.e. k=13 when using 5×5 VDA) to obtain the k-th shift value $(\Delta x, \Delta y)_k$:

$$(\Delta x, \Delta y)_k = \arg\max_{(x,y)} \mathcal{F}^{-1}\left\{\frac{\mathcal{F}\{I_{\text{center}}\} \odot \mathcal{F}^*\{I_k\}}{\left|\mathcal{F}\{I_{\text{center}}\} \odot \mathcal{F}^*\{I_k\}\right|}\right\}. \tag{S37}$$

Where $\mathcal{F}$ and $\mathcal{F}^{-1}$ denote Fourier and inverse Fourier transform, respectively. $\odot$ represents Hadamard product, and superscript asterisk * denotes complex conjugation. Bicubic interpolation was used to perform sub-pixel translational transformations on the image.

b. Superposition and postprocess.

After shifting the sub-images to align them with the center sub-image, the reconstruction result is obtained by directly stacking them, whose pixel size is equal to the scanning step size. The reconstruction result is then resized to the original image size by upsampling. Due to calculation precision errors, tilt DMD placement, and aberrations, there may be slight differences between pitch_x and pitch_y. Furthermore, there is a difference between the cumulative displacement $\text{pitch}_{\text{fast}}$ or $\text{pitch}_{\text{slow}}$ after moving 8 steps (i.e. a full scanning distance along the fast-axis direction or slow-axis direction) and $||\mathbf{v}_x||$ or $||\mathbf{v}_y||$ (the actual excitation lattice grid spacing). These deviations are corrected using scale-invariant feature transform[8] (setting affine transformation as expected transformation for the geometric consensus filter).

Note S5. Simulation of the effect of SD on DMD masks and C^2SD-ISM imaging

We use MATLAB to simulate the effect of SD on C^2SD-ISM imaging. When all DMD pixels are set to ON state, and the SD is removed, the C^2SD-ISM system degrades to a wide-field imaging system. Let the spatial distribution of the samples be denoted as Obj, and the detection PSF of the system PSF_{det} is generated based on the Born & Wolf PSF model[9]. In this case, the wide-field image can be obtained as:

$$\begin{aligned} I_{\text{WF}}(x,y,z) &= \text{Obj}(x,y,z) \otimes_{3D} \text{PSF}_{\text{det}}(x,y,z) \\ &= \mathcal{F}^{-1}\left\{\mathcal{F}\{\text{Obj}\} \cdot \mathcal{F}\{\text{PSF}_{\text{det}}\}\right\}(x,y,z). \end{aligned} \tag{S38}$$

Where $\otimes_{3D}$ denotes the three-dimensional convolution operation, $\mathcal{F}$ and $\mathcal{F}^{-1}$ represent the forward and inverse fast Fourier transforms, respectively.

After introducing the SD, the system is a confocal imaging system. According to (S1), the confocal image can be obtained as:

$$I_{\text{confocal}} = \text{Obj}(x,y,z) \otimes_{3D} \text{PSF}_{\text{confocal}}(x,y,z)\,, \tag{S39}$$

$$\text{PSF}_{\text{confocal}} = \text{PSF}_{\text{ex}}(x,y,z) \cdot \left(\text{PSF}_{\text{det}}(x,y,z) \otimes_{3D} A_{\text{det}}(x,y,z)\right). \tag{S40}$$

To investigate the effect of introducing SD on the super-resolution imaging performance of C^2SD-ISM, we simulated the modulation of the sample by structured illumination followed by detection, as follows:

$$I_i = \left[\text{Obj}(x,y,z) \cdot \mathcal{I}_i(x,y,z)\right] \otimes_{3D} \text{PSF}_{\text{det/confocal}}(x,y,z). \tag{S41}$$

$$\mathcal{I}_i(r,z) = \left[A_{\text{ex}}(x,y,z) \otimes_{3D} \text{PSF}_{\text{ex}}(x,y,z)\right] \otimes_{2D} \mathcal{L}_i(x,y,z). \tag{S42}$$

Where $\mathcal{I}$ denotes the illumination pattern, and the subscript i represents the i-th raw frame. If PSF_{det} is used, it signifies that the optical system is configured without the SD (w/o SD). If $\text{PSF}_{\text{confocal}}$ is used, the optical system is configured with the SD (w/ SD). $\mathcal{L}$ represents the position of the illumination point in three-dimensional space. When z corresponds to a position away from the focal plane, the matrix is a zero matrix. When z is at the focal plane, the matrix has a value of 1 at the illumination point position and 0 elsewhere.

We generated a series of square lattices to simulate DMD masks with different spacing (pixel size: 54 nm, matching the actual DMD pixel projection size under a 100× objective; Fig. S9a). By convolving the PSF_{ex} with these masks, the illumination patterns $\mathcal{I}$ are generated (Fig. S9b). It can be observed that smaller-spacing masks

lead to significant crosstalk during illumination, which reduces imaging contrast. We used the fluorescent plate shown in Fig. S9c as the sample function, with its thickness adjustable. For an animated demonstration, please refer to Movie S1.

Two 3D synthetic dataset, microtubules[10] and a cube of spherical beads as ground truth are used for simulating the effect of SD on C^2SD-ISM imaging. The synthetic microtubules is a 512×256×128 tif format image stack, which is publicly available on the website *https://bigwww.epfl.ch/deconvolution/*, along with its theoretical PSF. This synthetic dataset is well-suited for visually demonstrating the imaging effects under different optical configurations.

Additionally, to quantitatively assess the performance of different modalities, we generated our own cube of spherical beads as ground truth. As shown in Fig S11, this cube is a 256×256×1200 tif format image stack, consisting of a three-dimensional arrangement of identical spheres. In the x-direction, the sphere spacing starts at 300 nm and decreases stepwise by 20 nm down to 80 nm. In the y-direction, the spheres are spaced evenly at 200 nm intervals, while in the z-direction, the spacing starts at 700 nm and decreases stepwise by 20 nm down to 200 nm. The gray-scale variation of the spheres follows a Gaussian function, given by:

$$\text{bead}(x,y,z)=\exp\left\{-\frac{x^2+y^2+z^2}{2\sigma^2}\right\} \quad \sigma=\frac{\text{FWHM}}{2\sqrt{2\ln 2}}/\text{pixelsize}. \tag{S43}$$

The stimulation result is shown in Fig S11b and S11c, which demonstrate that the SD effectively removes defocused signals and significantly enhances both lateral and axial resolution, enabling superior DPA-PR reconstruction. Note that without the SD, the axial direction becomes indistinguishable, appearing as a straight line. Brightness differences arise due to variations in the spatial density of the beads, resulting in different energy distributions. To compare super-resolution results with GT images quantitatively, SSIM and PSNR metrics are used to applied to two-dimensional images extracted from the bead layer after preprocessing the wide-field raw data with the SD or different background removal algorithms. The results show that SD-DPA-PR achieves the highest consistence with the GT image, maintaining consistent high performance even at greater depths with more defocused signals (Fig S11d and S11e).

Note S6. Comparison of ISM Techniques Based on Digital Reconstruction

C^2SD-ISM achieves ISM-based super-resolution imaging by integrating multifocal excitation, confocal detection, and digital reconstruction. To highlight its distinction from other digitally reconstructed ISM techniques, the key differences are summarized in Table S1, followed by a detailed comparison of their respective technical implementations.

- **C^2SD-ISM** employs the DPA-PR algorithm, which prioritizes reconstruction fidelity while achieving resolution enhancement comparable to that of PR. The SD in C^2SD-ISM physically removes out-of-focus signals, which not only enhances axial resolution and increases imaging depth, but more importantly, significantly reduces crosstalk between adjacent foci under multifocal excitation. This reduction in crosstalk allows for denser multifocal patterns to be used, thereby lowering the number of raw frames required and effectively improving imaging speed. Moreover, leveraging the flexible programmability of the DMD, we have also implemented a SIM) modality.
- **MC-ISM** employs a pinhole array to generate multifocal excitation, combined with galvo-based scanning and algorithmic defocus removal. It typically requires 49 raw frames. As the sample thickness increases, to suppress optical crosstalk between adjacent foci, a pinhole array with a higher spacing-to-diameter ratio must be used. However, this adjustment decreases imaging speed, because requiring more raw frames to maintain full field-of-view coverage.
- **MSIM** employs a DMD-based binary mask to spatially modulate the illumination pattern. To minimize crosstalk between adjacent excitation spots, the mask must maintain a sparse distribution of "ON" pixels, which reduces excitation efficiency and necessitates a larger number of raw frames for reconstruction. Additionally, due to its wide-field detection configuration, MSIM exhibits relatively limited imaging depth compared to confocal-based systems.
- **CSD-ISM** leverages stroboscopic imaging by capturing multifocal snapshots under ultra-short exposure times during the SD rotation. To meet the Nyquist sampling requirement for the scanned illumination pattern, many raw frames must be acquired.
- **SPAD-ISM** employs a physical detector array, thus independent confocal sub-images are directly acquired, eliminating the need for VDA resampling as in C^2SD-

ISM. Only final sub-image registration and superposition are required, making the reconstruction algorithm comparatively simpler.

Table S1. Comparison of ISM techniques based on digital reconstruction.[a]

Technology	Lateral Res. (nm)	Axial Res. (nm)	Rec. method (Raw frames)	Speed[b]	Imaging depth[b]	Multimodality
**C^2SD-ISM**	144	351	DPA-PR (36/9)	★★★★	★★★★	SDCM /ISM /SIM
**MC-ISM** (Ren, W, et al. 2024. NSR 11.9: nwae303)	131	336	PR (49)	★★★	★★★	ISM
**MSIM** (York, A. G., et al. 2012. Nat. Methods 9, 749-754)	145	400	PR (224)	★★	★★	ISM
**CSD-ISM** (Schulze, O., et al. 2013. PNAS 110, 21000-21005)	130		PR (250)	★★	★★★	SDCM /ISM
**SPAD-ISM** (Castello, M., et al. 2019. Nat. Methods 16, 175-178)	193 ($\sqrt{2}\times$)		APR (Single point scan)	★★	★★★	CLSM /ISM

[a]All the data mentioned above are sourced from research papers.

[b]The more ★, the faster the system (or the deeper the imaging depth).

References

1 Zunino, A. *et al*. Open-source tools enable accessible and advanced image scanning microscopy data analysis. *Nature Photonics* **17**, 457-458 (2023).

2 Ströhl, F. & Kaminski, C. F. A joint Richardson-Lucy deconvolution algorithm for the reconstruction of multifocal structured illumination microscopy data. *Methods and applications in fluorescence* **3**, 014002 (2015).

3 York, A. G. *et al*. Resolution doubling in live, multicellular organisms via multifocal structured illumination microscopy. *Nature Methods* **9**, 749-754 (2012).

4 Ren, W. *et al*. Expanding super-resolution imaging versatility in organisms with multi-confocal image scanning microscopy. *National Science Review* **11**, nwae303 (2024).

5 Li, M. Q. et al. Structured illumination microscopy using digital micro-mirror device and coherent light source. *Applied Physics Letters* **116**, 233702 (2020).

6 Scholes, S., Kara, R., Pinnell, J., Rodríguez-Fajardo, V. & Forbes, A. Structured light with digital micromirror devices: a guide to best practice. *Optical Engineering* **59**, 041202-041202 (2020).

7 Guizar-Sicairos, M., Thurman, S. T. & Fienup, J. R. Efficient subpixel image registration algorithms. *Optics letters* **33**, 156-158 (2008).

8 Meer, P., Mintz, D., Rosenfeld, A. & Kim, D. Y. Robust regression methods for computer vision: A review. *International journal of computer vision* **6**, 59-70 (1991).

9 Born, M. & Wolf, E. Principles of optics: electromagnetic theory of propagation, interference and diffraction of light. 7th edn. (Elsevier, 2013).

10 Sage, D. et al. DeconvolutionLab2: An open-source software for deconvolution microscopy. *Methods* **115**, 28-41 (2017).
